# H2A.Z Acidic Patch Couples Chromatin Dynamics to Regulation of Gene Expression Programs during ESC Differentiation

**DOI:** 10.1371/journal.pgen.1003725

**Published:** 2013-08-22

**Authors:** Vidya Subramanian, Aprotim Mazumder, Lauren E. Surface, Vincent L. Butty, Paul A. Fields, Allison Alwan, Lillian Torrey, Kevin K. Thai, Stuart S. Levine, Mark Bathe, Laurie A. Boyer

**Affiliations:** 1 Department of Biology, Massachusetts Institute of Technology, Cambridge, Massachusetts, United States of America; 2 Laboratory for Computational Biology and Biophysics, and Department of Biological Engineering, Massachusetts Institute of Technology, Cambridge, Massachusetts, United States of America; 3 BioMicro Center, Massachusetts Institute of Technology, Cambridge, Massachusetts, United States of America; University of California San Francisco, United States of America

## Abstract

The histone H2A variant H2A.Z is essential for embryonic development and for proper control of developmental gene expression programs in embryonic stem cells (ESCs). Divergent regions of amino acid sequence of H2A.Z likely determine its functional specialization compared to core histone H2A. For example, H2A.Z contains three divergent residues in the essential C-terminal acidic patch that reside on the surface of the histone octamer as an uninterrupted acidic patch domain; however, we know little about how these residues contribute to chromatin structure and function. Here, we show that the divergent amino acids Gly92, Asp97, and Ser98 in the H2A.Z C-terminal acidic patch (H2A.Z^AP3^) are critical for lineage commitment during ESC differentiation. H2A.Z is enriched at most H3K4me3 promoters in ESCs including poised, bivalent promoters that harbor both activating and repressive marks, H3K4me3 and H3K27me3 respectively. We found that while H2A.Z^AP3^ interacted with its deposition complex and displayed a highly similar distribution pattern compared to wild-type H2A.Z, its enrichment levels were reduced at target promoters. Further analysis revealed that H2A.Z^AP3^ was less tightly associated with chromatin, suggesting that the mutant is more dynamic. Notably, bivalent genes in H2A.Z^AP3^ ESCs displayed significant changes in expression compared to active genes. Moreover, bivalent genes in H2A.Z^AP3^ ESCs gained H3.3, a variant associated with higher nucleosome turnover, compared to wild-type H2A.Z. We next performed single cell imaging to measure H2A.Z dynamics. We found that H2A.Z^AP3^ displayed higher mobility in chromatin compared to wild-type H2A.Z by fluorescent recovery after photobleaching (FRAP). Moreover, ESCs treated with the transcriptional inhibitor flavopiridol resulted in a decrease in the H2A.Z^AP3^ mobile fraction and an increase in its occupancy at target genes indicating that the mutant can be properly incorporated into chromatin. Collectively, our work suggests that the divergent residues in the H2A.Z acidic patch comprise a unique domain that couples control of chromatin dynamics to the regulation of developmental gene expression patterns during lineage commitment.

## Author Summary

Elucidating how regulation of chromatin structure modulates gene expression patterns is fundamental for understanding mammalian development. Replacement of core histones with histone variants has recently emerged as a key mechanism for regulating chromatin states. The histone H2A variant H2A.Z is of particular interest because it is essential for embryonic development and for proper execution of developmental gene expression programs during cellular specification. ESCs provide a good model for investigating the function of H2A.Z during lineage commitment because these cells can generate an unlimited number of equivalent descendants while maintaining the capacity to differentiate into any cell type in the organism. Divergent regions in H2A.Z are likely key for functional specialization, but we know little about how these differences contribute to chromatin regulation. Here, we show that the unique H2A.Z acidic patch domain is necessary for regulation of lineage commitment during ESC differentiation by linking transcription to chromatin dynamics. Our work provides a critical foundation for elucidating how H2A.Z incorporation is key to cell fate determination. These findings are particularly important given that H2A.Z has been implicated in many diseased conditions, including cancer.

## Introduction

Precise control of gene expression is critical for lineage commitment and proper development in all multicellular organisms. Regulation of chromatin structure has emerged as an important mechanism for modulating gene expression patterns in response to developmental cues. While post-translational histone modifications can influence chromatin structure and transcriptional activity, less is known about the role of histone variants. Histone variants are incorporated in a replication-independent manner and appear to mark structurally and functionally distinct chromatin domains [1]–[3]. The histone H2A variant H2A.Z is highly conserved among eukaryotes and is of particular interest because it plays an essential but poorly understood role in metazoan development including mammals [4]–[6]. H2A.Z has been implicated in a range of DNA-mediated processes such as gene expression, DNA repair, and genomic stability [7]–[9]. Notably, H2A.Z is required for proper execution of developmental gene expression programs during embryonic stem cell (ESC) differentiation [10], suggesting that H2A.Z has specialized functions to regulate lineage commitment.

A role for H2A.Z in gene regulation is supported by genome-wide localization studies showing that this variant flanks the nucleosome-free region at transcription start sites in a wide range of cell types [11], [12]. In particular, H2A.Z is incorporated at the majority of H3K4me3 modified promoter nucleosomes including bivalent promoters in ESCs that harbor both H3K4me3 and H3K27me3, marks of Trithorax and Polycomb, respectively [10], [11]. Bivalent promoters in ESCs are associated with lineage specific genes that are poised, but remain competent for activation [13], [14]. These studies suggest that H2A.Z contributes to formation of distinct chromatin states and that its incorporation at bivalent promoters may be necessary to allow for induction of lineage programs in response to developmental cues. Consistent with this idea, H2A.Z levels decreased at promoters upon gene activation [11], [15]–[17]. H2A.Z also occupied regulatory elements such as enhancers and boundary elements [11], [18], [19], indicating that H2A.Z is incorporated at regions that are subject to considerable chromatin regulation.

H2A.Z shares extensive homology with the major type histone H2A throughout the histone fold domain. However, divergent regions in the amino- and carboxy-terminal domains as well as the L1 loop region within the histone fold suggest that the two histones have different structural and functional properties. *In vitro* biophysical studies showed that H2A.Z incorporation stabilizes the dimer-tetramer interface and strongly favors formation of 30 nm fibers over formation of higher order chromatin folding that require fiber-fiber interactions when compared to canonical H2A, consistent with the idea that unique features of the variant contribute to specialized chromatin domains [20]–[22]. Moreover, H2A.Z and the histone H3 variant H3.3 can occupy the same nucleosome resulting in a double variant nucleosome that is enriched at active promoters as well as at highly regulated chromatin regions [23], [24]. These hybrid nucleosomes are characterized as unstable and highly salt labile, consistent with its presence in dynamic chromatin domains [25], [26]. Thus, dissecting the features of H2A.Z that distinguish it from core H2A is key to understanding its functional specialization and may provide new insights into the essential role of this variant during development.

The H2A.Z carboxy-terminal acidic patch comprises substitutions of H2A residues Asn89, Asn94, Lys95 for Gly92, Asp97, and Ser98. While the overall structure of the H2A.Z nucleosome appears similar to H2A-containing nucleosomes, these three divergent residues form an uninterrupted acidic patch that extends across the surface of the H2A.Z octamer resulting in a solvent-exposed cavity in the center of the nucleosome [27]. In *Drosophila*, domain-swap experiments demonstrated that the H2A.Z carboxy-terminus including the acidic patch is essential for development [28]. Later work in Xenopus involving site-specific mutagenesis of the divergent H2A.Z acidic patch residues resulted in embryos that exhibited significant developmental defects [6]. These studies suggest that the unique H2A.Z acidic patch plays an important role in establishing a novel chromatin state that is essential for embryonic development. In yeast, replacement of two residues in the H2A.Z acidic patch resulted in low nucleosome occupancy at the *PHO5* promoter, suggesting that the acidic patch is necessary for interaction with its deposition complex and for proper incorporation into chromatin [29]. However, the amino acids mutated in this study are conserved between H2A.Z and canonical H2A, indicating that this phenotype is not likely specific to H2A.Z. Other loss-of-function studies in yeast have shown that the H2A.Z carboxy-terminal docking domain is critical for H2A.Z function; however, these studies examined truncations that retained an intact acidic patch domain [30], [31]. Thus, we currently lack a detailed mechanistic understanding of how this domain regulates chromatin conformation during development and whether it plays a similar role in mammals.

We investigated the role of the divergent H2A.Z acidic patch during ESC differentiation. ESCs are an ideal model for investigating how H2A.Z influences mammalian development because these cells maintain the potential to differentiate into all somatic cell types [32], [33]. We generated a mutant form of H2A.Z (denoted H2A.Z^AP3^), where the three divergent acidic patch residues in H2A.Z are replaced with the corresponding H2A amino acids. H2A.Z^AP3^ ESCs maintained the ability to self-renew, but these cells failed to differentiate properly. We found that while H2A.Z^AP3^ interacted with its deposition complex and displayed a highly similar distribution pattern compared to expression of the wild-type H2A.Z transgene (denoted H2A.Z^WT^), its enrichment levels were reduced at target promoters and were particularly diminished at the +1 nucleosome. Further analyses revealed that H2A.Z^AP3^ was less tightly associated with chromatin compared to H2A.Z^WT^ suggesting that the mutant is more dynamic. Notably, bivalent genes that are poised for activation in ESCs displayed significant changes in expression compared to active genes suggesting that the poised state is more sensitive to H2A.Z regulation. Moreover, this group of genes showed reduced levels of the repressive chromatin mark H3K27me3 at H2A.Z^AP3^ target gene promoters compared to H2A.Z^WT^ and displayed higher H3.3 enrichment, a variant associated with high chromatin flux. Consistent with this observation, we further showed that H2A.Z^AP3^ displayed higher mobility in chromatin compared to H2A.Z^WT^ by fluorescence recovery after photobleaching (FRAP). Remarkably, ESCs treated with the transcriptional inhibitor flavopiridol partially restored the H2A.Z^AP3^ mobile fraction to wild-type levels and resulted in an increase in H2A.Z^AP3^ occupancy at target genes. Collectively, our results demonstrate that the divergent H2A.Z acidic patch mediates chromatin dynamics and indicate that control of H2A.Z dynamics is critical for the regulation of gene expression patterns during lineage commitment.

## Results

### H2A.Z acidic patch is necessary for ESC differentiation

**Figure 1:**
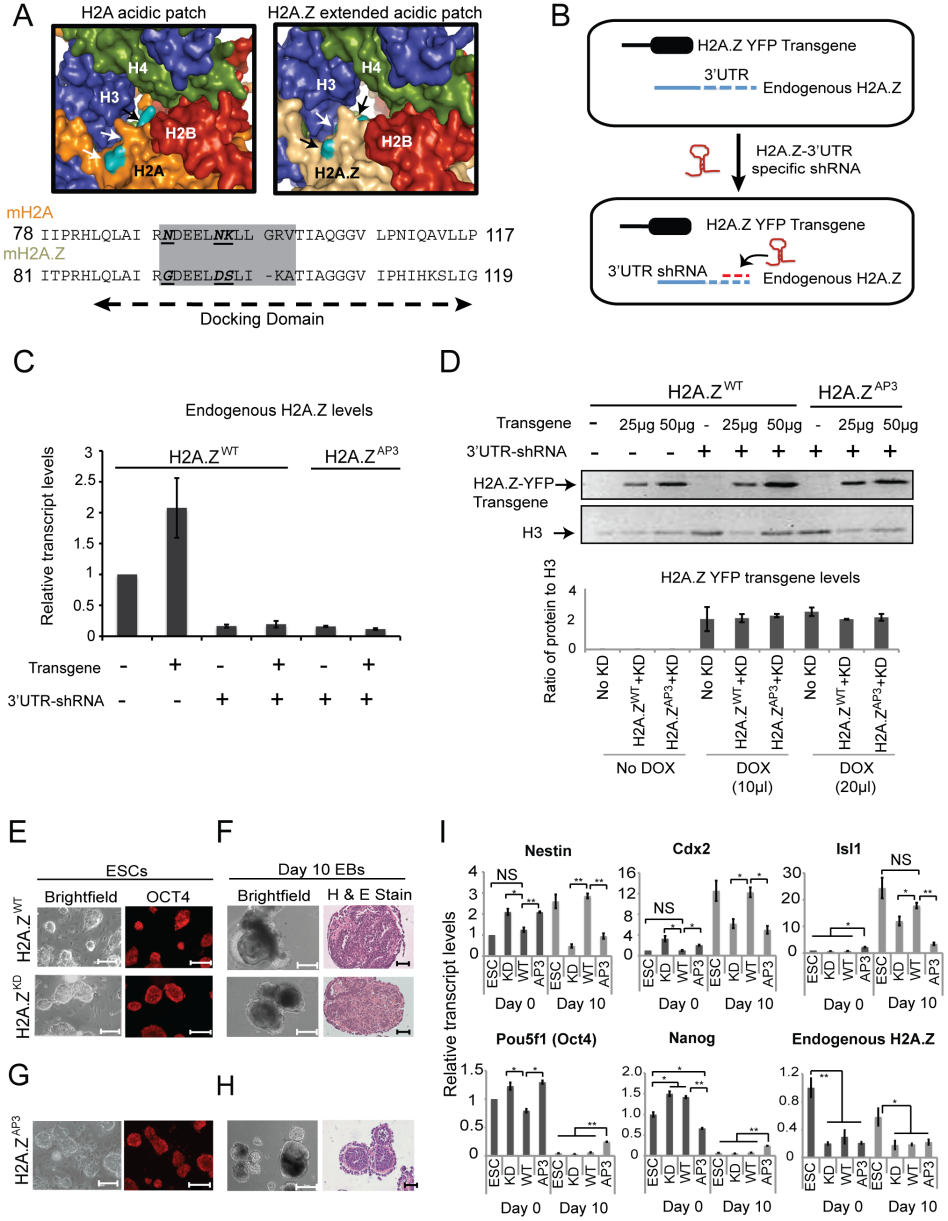
H2A.Z acidic patch is necessary for ESC differentiation. (A) Surface rendering of the H2A (left) and H2A.Z (right) nucleosome center. The H2A (orange) and H2A.Z (light brown) structures are shown with H2B (red), H3 (blue), and H4 (green) as indicated by the labels. The divergent residues (teal) are highlighted with arrows. The images were generated in Pymol using the following PDB files: 1AOI for canonical H2A-containing nucleosome structure and 1F66 for H2A.Z nucleosome structure. Below, sequence alignment of C terminal docking domain of H2A and H2A.Z. The acidic patch region is highlighted in a grey box. The bold, italicized and underlined residues indicate the divergent H2A.Z residues replaced in our study to the corresponding H2A residues. (B) Schematic diagram depicting the system used in this study to investigate the function of the H2A.Z acidic patch. (C) qRT-PCR representing the relative levels of endogenous H2A.Z transcript in H2A.Z^WT^ and H2A.Z^AP3^ dox-inducible transgenic ESCs in the presence (+) and absence (−) of H2A.Z 3′UTR-specific shRNA. Transcript levels were normalized relative to Tubb5. Error bars represents standard deviation calculated from three independent biological replicates. (D) (Top) Western blot using H2A.Z antibodies on whole cell lysates isolated from dox-induced and uninduced (−/+) H2A.Z^WT^ and H2A.Z^AP3^ transgenic ESC lines in the presence (+) and absence (−) of the H2A.Z 3′UTR-specific shRNA. Titrations of the dox-induced samples (25 µg and 50 µg of whole cell lysates) were performed to demonstrate comparable expression of H2A.Z-YFP transgene in H2A.Z^WT^ and H2A.Z^AP3^ ESCs. H3 levels were used as a load control (lower exposure). Densitometric measurements were used to determine the ratio of transgene signal to H3 for the indicated samples (bottom) using ImageJ. Error bars represent standard deviations from a triplicate set of experiments. ESC colony morphology and OCT4 staining for H2A.Z^WT^, H2A.Z^KD^, (E) and H2A.Z^AP3^ (G) expressing ESC. Embryoid bodies (EBs) were generated from H2A.Z^WT^, H2A.Z^KD^ (F), and H2A.Z^AP3^ (H) expressing ESCs. The left panels show bright field images of EBs cultured for 10 days in the absence of LIF. The right panels show hematoxylin and eosin stained sections of day 10 EBs for indicated cell lines. (I) qRT-PCR analyses showing mRNA levels of indicated pluripotency (*Pou5f1/Oct4* and *Nanog*) and differentiation markers (*Nestin*, *Cdx2*, *Isl1*) in Day 0 and Day 10 EBs generated from unmodified ESCs, H2A.Z^WT^, H2A.Z^KD^, and H2A.Z^AP3^ ESCs. Student's t-test was performed to generate indicated p values. ***p<0.005, **p<0.01, *p<0.05. Error bars represent standard deviations from a triplicate set of experiments.

The H2A acidic patch domain resides on the nucleosome surface, and in the case of H2A.Z contains three divergent residues that comprise an extended acidic patch [27] (Figure 1A). While disruption of the H2A.Z acidic patch results in early developmental defects in *Drosophila* and *Xenopus*, how it functions to regulate chromatin structure and whether this domain plays a similar role in mammals is unknown [6], [28]. In mouse, H2A.Z is encoded by two isoforms that differ by only 3 amino acid residues, denoted H2AFZ (H2A.Z) and H2AFV [34], [35] (Figure S1A). H2A.Z knockout mice die around the time of implantation [5], suggesting that H2AFV is unable to compensate for loss of H2A.Z and that the two isoforms are functionally distinct. We analyzed the relative abundance of H2A.Z and H2AFV in ESCs by mass spectrometry. While we detected both isoforms in ESCs, H2A.Z is ∼20-fold more abundant than H2AFV (Figure S1B). Thus, given the essential role of H2A.Z and its abundance in ESCs compared to H2AFV, we focused on dissecting the function of the divergent acidic patch in H2A.Z.

Since H2A.Z and H2AFV share nearly identical amino acid sequences, available antibodies cannot distinguish between the two isoforms. To circumvent this limitation, we generated ESC lines that harbor a stably integrated Tet-inducible H2A.Z transgene fused to YFP (Figure 1B). Upon induction by doxycycline, we sorted for YFP expression and collected cells that displayed transgene expression comparable to endogenous H2A.Z levels for further analysis. To specifically test transgene function, we integrated short hairpins directed against the H2A.Z 3′ UTR into the inducible ESC lines (Figure 1B). This system allowed for targeted depletion of endogenous H2A.Z (denoted H2A.Z^KD^ for knock-down) (Figure 1C, Figure S1D) without affecting transgene levels or H2AFV expression (Figure 1D, Figure S1C). Consistent with our previous work, H2A.Z^KD^ ESCs displayed typical colony morphology, cell cycle kinetics, and normal expression of the pluripotency marker *Pou5f1* (*Oct4*) suggesting that H2A.Z depletion does not affect self-renewal (Figure 1E, Figure S1E, F). To test differentiation potential, wild-type H2A.Z-YFP (denoted H2A.Z^WT^) and H2A.Z^KD^ ESCs were induced to differentiate by allowing these cells to aggregate into embryoid bodies (EBs) in the absence of the pluripotency growth factor LIF. EBs are similar to egg cylinder stage embryos, albeit disorganized, and are capable of differentiation into tissues representing the three germ layers. H2A.Z^KD^ ESCs failed to differentiate properly, and lacked the distinct differentiated structures observed in Day 10 EBs compared to H2A.Z^WT^ (Figure 1F). Additionally, EBs from H2A.Z^KD^ ESCs failed to activate developmental genes to levels observed in H2A.Z^WT^ cells (Figure 1I), consistent with the idea that H2A.Z regulates lineage programs [11]. Importantly, expression of H2A.Z^WT^ rescued the H2A.Z^KD^ phenotype as measured by the restoration of ESC gene expression patterns as well as their capacity for multi-lineage differentiation (Figure 1F,I), whereas H2A-YFP did not compensate for loss of H2A.Z (data not shown). These data indicate that expression of the H2A.Z^WT^ transgene recapitulates normal H2A.Z function. Thus, we used this system to dissect the role of the H2A.Z acidic patch during lineage commitment.

We replaced the divergent H2A.Z acidic patch residues by site-directed mutagenesis of Gly92, Asp97, and Ser98 to the equivalent residues in H2A- Asn89, Asn94, and Lys95 (Figure 1A). Similar to H2A.Z depletion, expression of the acidic patch mutant (denoted H2A.Z^AP3^) in H2A.Z^KD^ ESCs did not affect self-renewal, colony morphology, or levels of the pluripotency marker OCT4 (Figure 1G). We observed, however, that H2A.Z^AP3^ EBs were smaller, morphologically distinct, and failed to differentiate properly compared to H2A.Z^WT^ EBs as demonstrated by the lack of differentiated cell types at Day 10 and the inability to activate developmental gene expression programs during lineage commitment (Figure 1H,I). The smaller size of H2A.Z^AP3^ EBs was not a result of altered cell cycle kinetics, proliferation, or differences in levels of apoptotic cells relative to H2A.Z^WT^ (Figure S1E–I). Moreover, the number of cells recovered from Day 10 H2A.Z^AP3^ EBs was comparable to that recovered from H2A.Z^WT^, and H2A.Z^KD^ EBs (Figure S1J). Rather, we observed a larger number of small EBs in H2A.Z^AP3^ cultures compared to H2A.Z^WT^. Taken together, these data suggest that the divergent residues in the H2A.Z acidic patch are necessary for proper ESC differentiation.

### H2A.Z acidic patch mutant is enriched at typical H2A.Z target genes

**Figure 2:**
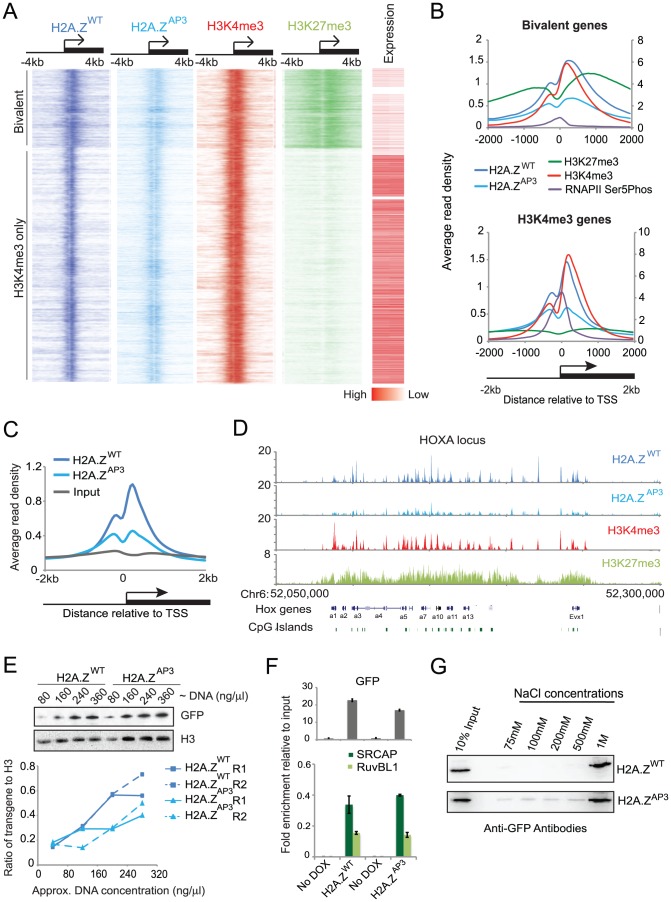
H2A.Z acidic patch is incorporated at lower levels at target genes. ChIP-Seq analysis of H2A.Z in ESCs shows that the divergent acidic patch residues are required for stable incorporation of H2A.Z (A) Density map of H2A.Z^WT^ (dark blue), H2A.Z^AP3^ (light blue), H3K4me3 (red), and H3K27me3 (light green) enrichment at all H2A.Z target genes ordered from most H3K27me3 enriched genes to least H3K27me3 enriched genes in ESCs within the region −4 kb to +4 kb relative to the TSS. The right panel representing the expression levels of the corresponding genes in ESCs generated from RNA-Seq data. Red to white gradient represents genes with high to low expression levels respectively. (B) Average enrichment patterns of H2A.Z^WT^, H2A.Z^AP3^, H3K4me3, H3K27me3, and RNAP2-Ser5P +/−2 kb around the TSS at bivalent (top) and H3K4me3 (H3K27me3 negative) only promoters (bottom). H2A.Z^WT^, H2A.Z^AP3^, and H3K27me3 are plotted on the primary axis (right). H3K4me3 and RNAP2-Ser5P are plotted on the secondary axis (left). (C) Average read density plots comparing binding profiles of H2A.Z^WT^, H2A.Z^AP3^, and input at all H2A.Z target gene promoters in ESCs plotted +/−2 kb relative to TSS. The ChIP-Seq datasets for H2A.Z^WT^ and H2A.Z^AP3^ were generated using GFP antibodies against the YFP transgene. (D) Genome profile of ChIP-Seq reads showing the distribution of H2A.Z^WT^ (dark blue), H2A.Z^AP3^ (light blue), H3K4me3 (red), and H3K27me3 (light green) across the HoxA locus- a representative set of H2A.Z target genes. (E) Semi-quantitative western blot of H2A.Z^WT^ and H2A.Z^AP3^ chromatin fractions probed with GFP and H3 (load control) using a range of DNA concentrations (top). Graph quantifying the ratio of transgene levels relative to H3 at the indicated DNA concentrations shows ∼1.85 fold more H2A.Z^WT^ in chromatin fractions compared to H2A.Z^AP3^ (bottom). Fold change was calculated from the average ratio of each transgene to H3. Ratios for H2A.Z^WT^/H3 (0.439) and H2A.Z^AP3^/H3 (0.255) at the two intermediate DNA concentrations (160 µg/µl and 240 µg/µl) for replicate 1 (R1) were used to calculate the 1.72 (0.439/0.255) fold change between H2A.Z^WT^ and H2A.Z^AP3^. Similar results were obtained for an independent replicate (R2). Ratios for H2A.Z^WT^/H3 (0.439) and H2A.Z^AP3^/H3 (0.219) at the two intermediate DNA concentrations (160 µg/µl and 240 µg/µl) were used to calculate the 2.0 (0.439/0.219) for R2. Thus, the levels of H2A.Z^WT^ were on average 1.85-fold higher in chromatin-associated fractions relative to H2A.Z^AP3^. (F) Graph showing the ratio of SRCAP and RUVBL1 signal to their respective input signal, from co-immunoprecipitation analyses performed in H2A.Z^WT^ and H2A.Z^AP3^ ESCs (in the endogenous H2A.Z knockdown background). Densitometric measurements of the western blots were performed in ImageJ. The standard deviations were generated from triplicates data points. (G) Nuclei isolated from H2A.Z^WT^ and H2A.Z^AP3^ expressing ESCs were subjected to increasing salt concentrations as indicated. Histones were extracted at these salt concentrations and resolved by SDS-PAGE. Histones were detected by immunoblotting with GFP antibodies.

H2A.Z containing nucleosomes occupy majority of promoters from yeast to human as determined by genome-wide localization studies and its incorporation is critical for proper gene regulation [10], [11], [36]. Thus, we analyzed the localization pattern of the acidic patch mutant relative to H2A.Z. Given that current H2A.Z antibodies cannot distinguish between H2A.Z and H2AFV isoforms, we performed ChIP-Seq in ESCs using GFP antibodies that recognize the H2A.Z-YFP tag. This analysis revealed significant H2A.Z-specific enrichment at ∼11,000 promoters in ESCs (Table S1). We found by ChIP-Seq that H2A.Z enrichment overlapped with the majority of H3K4me3 promoters, a histone modification that is normally associated with transcriptional competence (Figure 2A) [11], [37]. Our H2A.Z data are similar to other genome wide reports using pan H2A.Z antibodies in ESCs [11], [36], indicating that the YFP tag does not affect H2A.Z incorporation.

In addition to its enrichment at active promoters, H2A.Z was enriched at bivalent promoters consistent with our prior ChIP-chip analysis [10]. Bivalent promoters are characterized by the enrichment of H3K4me3 and H3K27me3 marks, and these genes display low expression levels and reduced enrichment of RNA Polymerase II (RNAP2) (Figure 2A, B top) [13], [14]. In contrast, H2A.Z enrichment at active promoters (H3K4me3-only) was coincident with a strong RNAP2 peak at the TSS and higher expression of associated genes (Figure 2A, B bottom). Our analysis also revealed a bimodal H2A.Z distribution pattern around TSSs with a marked enrichment at the +1 nucleosome (Figure 2C), consistent with reports showing that H2A.Z flanks the nucleosome-depleted region [11]. Notably, we observed a broader distribution pattern of H2A.Z and H3K4me3 at bivalent genes compared to their enrichment at active genes suggesting that the chromatin structure differs at these two classes of promoters. In addition to promoters, we found that H2A.Z was enriched at a subset of distal enhancers identified in ESCs [38] (Table S1), similar to recent data using a pan-H2AZ antibody [36]. The enrichment at promoters as well as distal regulatory elements suggests that H2A.Z is incorporated at regions subject to considerable chromatin regulation.

We next analyzed H2A.Z^AP3^ localization across the ESC genome by ChIP-Seq. While H2A.Z^AP3^ enrichment was globally decreased, it occupied a highly similar set of promoter regions as well as distal enhancers (Figure 2C, Table S1). For example, H2A.Z^AP3^ displayed a similar overall spatial pattern of enrichment, albeit reduced compared to H2A.Z^WT^ as shown across the large bivalent region encompassing the HOXA locus in ESCs (Figure 2D). Importantly, H2A.Z^AP3^ was expressed at similar levels as H2A.Z^WT^ (Figure 1D, Figure S2A, B), indicating that reduced H2A.Z^AP3^ enrichment was not due to its lower abundance in ESCs. Interestingly, we observed a dramatic reduction in H2A.Z^AP3^ enrichment downstream of the TSS in ESCs, which is thought to mark the +1 nucleosome (Figure 2C). A number of studies have suggested that the +1 nucleosome possesses significant regulatory potential and that remodeling of this promoter nucleosome may be important for controlling gene expression by recruiting RNAP2 or by facilitating transcriptional elongation [39], [40]. Collectively, these data suggest that altered levels of H2A.Z^AP3^ as well as a particular reduction in the +1 nucleosome at promoters have consequences on the regulation of gene expression states.

One possibility for the observed lower levels of H2A.Z^AP3^ is that replacement of the divergent amino acids with those of H2A lead to its incorporation via a similar pathway as core histones. We expected that if H2A.Z^AP3^ is more broadly distributed along chromosomes, then its overall chromatin-associated fraction would be similar or higher relative to H2A.Z^WT^. To this end, we performed chromatin fractionation and probed for H2A.Z^WT^ and H2A.Z^AP3^ using GFP antibodies in the respective transgenic ESC line (Figure S2B). Semi-quantitative immunoblots showed that the fraction of H2A.Z^WT^ associated with chromatin is approximately 1.85 fold higher than H2A.Z^AP3^ suggesting that depletion of the mutant at TSSs does not lead to its random accumulation in chromatin (Figure 2E). Given recent evidence demonstrating that H2A.Z is redistributed during the cell cycle from promoters to heterochromatin regions in mouse trophoblast stem (TS) cells [41], next we examined the levels of H2A.Z^AP3^ at these regions. We first analyzed metaphase chromosomes (a time point when H2A.Z is enriched at heterochromatin in TS cells) and found that unlike the broad distribution of H2A across the chromosome H2A.Z^AP3^ ESCs showed a non-uniform distribution pattern and depletion at centromeric regions similar to H2A.Z^WT^ (Figure S2C). Consistent with this observation, we found both H2A.Z^AP3^ and H2A.Z^WT^ showed overall low enrichment at repetitive elements associated with heterochromatin including major satellite repeats in cycling cells (Figure S2D). Additionally, we found no change in the levels of H3K9me3, a modification highly enriched in heterochromatin, in H2A.Z^WT^ and H2A.Z^AP3^ ESCs as well as in Day 5 RA differentiated cells by ChIP-qPCR (Figure S2E). The lower levels of H2A.Z at heterochromatin regions in ESCs compared to TS cells may reflect differences in cell cycle dynamics between the two cell types, as ESCs have a notably short G1. Together, these data suggest that mutations in the H2A.Z acidic patch do not result in the inappropriate incorporation of H2A.Z^AP3^ or disruption of heterochromatin.

### H2A.Z acidic patch domain is necessary for stable chromatin association

The observed lower levels of H2A.Z^AP3^ at promoters suggested that the mutant is either more dynamically associated with chromatin or that it is not properly incorporated by its deposition complex. In yeast, deletion of either the carboxy-terminal docking domain or mutation of conserved residues within the acidic patch resulted in low H2A.Z occupancy at target genes [29]–[31]. This decrease in H2A.Z occupancy was attributed to the inability of the carboxy-terminal mutant to interact with its deposition complex SWR1. Site-specific incorporation of H2A.Z in mammalian cells is accomplished by the ATPase complex SRCAP (Snf2-related CREBBP activator protein), and in yeast H2A.Z removal appears to require the INO80 complex [42], [43]. Thus, we tested the ability of H2A.Z^AP3^ to interact with components of both of these complexes. We performed co-immunoprecipitation followed by immunoblot and found that H2A.Z^AP3^ interacted with SRCAP (catalytic component of SRCAP) and RUVBL1 (component of Tip60, SRCAP, and INO80 complexes) similar to H2A.Z^WT^ (Figure S2F, Figure 2F). While it is possible that H2A.Z^AP3^ renders the deposition complex catalytically inactive, this scenario is unlikely because endogenous H2A.Z enrichment at promoters was unchanged over multiple passages in cells that also expressed the H2A.Z^AP3^ transgene (Figure S2G).

While H2A.Z specific chaperones such as CHZ1 have been identified in yeast [44], the CHZ1 homolog or chaperones that play similar roles in mammalian cells have not been studied in detail. Nevertheless, NAP1 is also a critical chaperone for histone incorporation in mammals including H2A.Z so we sought to test whether alterations in the acidic patch affected its interaction with this histone chaperone. Thus, we quantified the interaction between NAP1L1 and H2A.Z^AP3^, H2A.Z^WT^, as well as core H2A (Figure S2H, I). We found by co-immunoprecipitation using GFP antibodies that the amount of NAP1L1 bound to H2A.Z^AP3^ was modestly higher (<2-fold) compared to H2A.Z^WT^ (Figure S2H, I). Surprisingly, we observed that lower levels of NAP1L1 co-immunoprecipitated with H2A compared to H2A.Z^WT^ (Figure S2H, I). This result may be due to the much higher levels of stably associated H2A in chromosomes, of which only a fraction of H2A would interact with NAP1L1 unlike the continuous dynamic replacement of H2A.Z. Thus, these data are consistent with the idea that H2A.Z^AP3^ is more dynamic, resulting in a higher fraction of the mutant available to interact with histone chaperones.

We next tested the idea that H2A.Z^AP3^ is less tightly associated with chromatin compared to H2A.Z^WT^. To this end, we performed salt titrations on nuclei isolated from H2A.Z^WT^ and H2A.Z^AP3^ ESCs. This analysis showed that while H2A.Z^WT^ was stably associated with chromatin up to 500 mM NaCl and largely depleted at 1M NaCl (dimer loss typically occurs between 600–800 nM NaCl), a fraction of H2A.Z^AP3^ dissociates at the lower salt concentrations (75 mM–200 mM NaCl) (Figure 2G), suggesting that H2A.Z^AP3^ is less tightly associated with chromatin. The finding that H2A.Z^AP3^ is less tightly associated with chromatin by salt titration is consistent with the higher proportion of H2A.Z^AP3^ associated with NAP1L1 in ESCs. While we cannot rule out that H2A.Z^AP3^ incorporation is less efficient or that a small fraction is randomly distributed similar to incorporation of H2A, our data are consistent with the model that the unique H2A.Z extended acidic patch is critical for regulating its dynamic association with chromatin. These data also suggest that control of H2A.Z dynamics is important for regulation of gene expression programs during lineage commitment.

### H2A.Z acidic patch is necessary for regulation of bivalent genes during ESC differentiation

**Figure 3:**
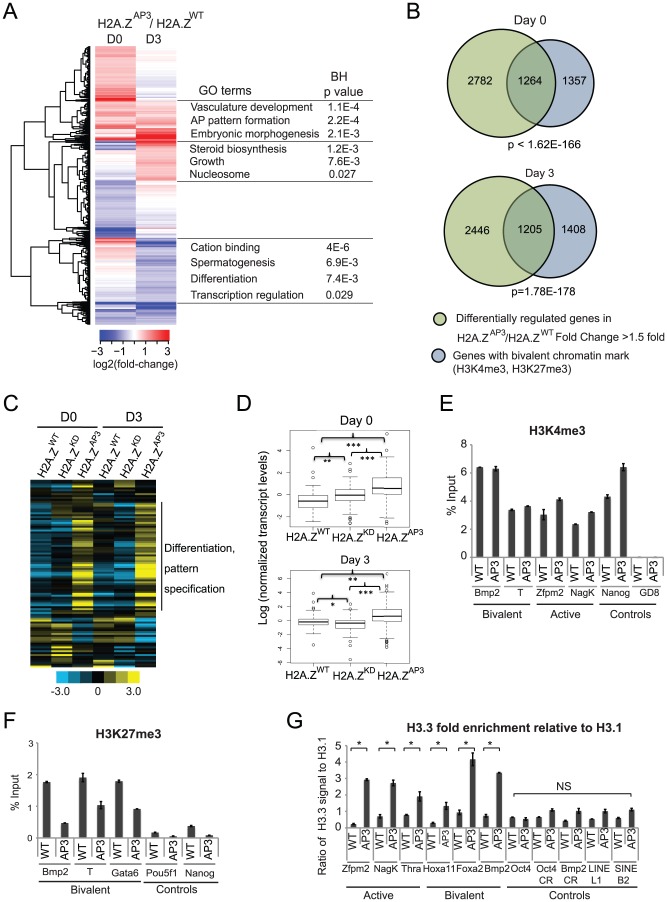
H2A.Z acidic patch is necessary for regulation of bivalent genes during ESC differentiation. (A) Heat map displaying genes with absolute fold-changes of >1.5 fold at D0 or D3 in H2A.Z^AP3^ EBs relative to H2A.Z^WT^. Log2-fold changes in expression of these genes were hierarchically clustered using a Euclidean distance metric. Gene Ontology analysis of the clusters was performed using DAVID. (B) Venn diagrams representing overlap of differentially-regulated genes (>1.5 fold) in H2A.Z^AP3^ relative to H2A.Z^WT^ (green) and genes with the bivalent chromatin mark within +/−2 kb of its TSS (blue) at D0 (top) and D3 (bottom). The p values were calculated by hypergeometric tests. (C) Heat map showing changes in transcript levels of a representative subset of genes (including developmental regulators, pluripotency factors, chromatin regulators and house keeping genes) using Nanostring in H2A.Z^WT^, H2A.Z^KD^, and H2A.Z^AP3^ ESCs at D0 and D3 of EB differentiation. The heat map was generated using log counts from each experiment, which were normalized to the geometric mean of the expression of housekeeping genes *Gapdh*, *Tubb5*, and *Cltc* (Table S3). Genes were then normalized across the 6 experiments. Yellow represents genes that are up-regulated 2 fold or over and blue represents genes down-regulated by 2 fold or more. (D) Boxplots representing the median expression levels of H2A.Z^WT^, H2A.Z^KD^, and H2A.Z^AP3^ D0 and D3 EBs were generated using log transformed, normalized gene expression values of all genes represented in the Nanostring probe set (Table S3). P values were calculated by unpaired student t-test. *p<0.008, **p<0.0008, and ***p<0.0002. The false discovery rate for a p value 0.0074 (<0.008) was generated using Monte Carlo simulation and found to be 3.9% from 10,000 random iterations, suggesting a small but significant change in median expression levels between Day 3 H2A.Z^WT^ and H2A.Z^KD^ EBs. qPCR was performed on ChIP DNA generated in H2A.Z^WT^ and H2A.Z^AP3^ ESCs using H3K4me3 (E) and H3K27me3 (F) antibodies. (G) Quantitative qPCR was performed on ChIP-DNA enriched for H3.3 and H3.1 (Flag-HA tagged) using a mixture of Flag and HA antibodies in H2A.Z^WT^ and H2A.Z^AP3^ containing HA-Flag tagged H3.3 and H3.1 transgenes independently. The ratio of H3.3 enrichment relative to H3.1 was plotted for active (*Zfpm2, NagK, Thra*) and bivalent (*Hoxa11, Foxa2, Bmp2*) H2A.Z target promoters. Relative enrichment ratios were also plotted for control regions- *Pou5f1/Oct4* promoter, coding regions for *Pou5f1* (Pou5f1-CR) and *Bmp2* (Bmp2-CR) and heterochromatin elements (LINE L1, SINE B2). Student's t-test was performed to generate indicated p values. *p<0.05. Error bars represent standard deviations from a triplicate set of experiments. NS indicates p values >0.5.

H2A.Z knock-out mice die around the time of gastrulation when complex gene expression patterns are established during embryogenesis [5]. Moreover, ESCs depleted of H2A.Z fail to execute developmental gene expression programs when signaled to do so [10]. Furthermore, *in vitro* biophysical studies showed that an intact H2A.Z acidic patch is necessary for the ability of a nucleosomal template to fold into a 30-nm chromatin fiber and for the efficient repression of transcription [45], [46]. Thus, we analyzed global gene expression patterns in H2A.Z^WT^ and H2A.Z^AP3^ cells in ESCs (Day 0 or D0) and at Day 3 (D3) of ESC differentiation (Table S2). We observed that a subset of genes showed higher expression levels in H2A.Z^AP3^ at D0 and that many of these genes remained expressed at higher levels in Day 3 H2A.Z^AP3^ EBs relative to H2A.Z^WT^ (Figure 3A). These de-repressed genes have functions in vasculature development, pattern formation, and embryonic morphogenesis (e.g. *Tbx20*, *Hoxb4*, *Foxc1*) (Figure 3A). Genes that displayed higher expression levels in H2A.Z^AP3^ specifically at D3, function in steroid biosynthesis, signaling, and growth (e.g. *Cyp51*, *Mvd*, *Wnt5a*). In contrast, genes with lower expression levels at D3 in H2A.Z^AP3^ have roles in differentiation and transcription regulation (e.g. *Notch4*, *Spag1*, *Neurod1*, *Wnt5a*). These results indicate that H2A.Z^AP3^ incorporation leads to significant changes in the expression of genes with important developmental functions.

While H2A.Z is enriched at the majority of H3K4me3 marked promoters, we previously observed that bivalent genes exhibited significant changes upon H2A.Z depletion [10]. In ESCs, H2A.Z incorporation at bivalent promoters is required for precise regulation of developmental programs during the initial stages of lineage commitment. Notably, genes that showed changes in H2A.Z^AP3^ ESCs comprised a large cohort of developmental regulators (Figure 3A). Thus, we further tested the connection between H2A.Z and the regulation of bivalent genes. We compared the differentially regulated genes in H2A.Z^AP3^ cells (D0 and D3) with genes containing either bivalent (H3K4me3 and H3K27me3) or active (H3K4me3 only) histone marks. We found a significant overlap between genes that are differentially regulated and genes with bivalent marks (P<10^−100^, Figure 3B), whereas no significant overlap was observed with H3K4me3 only (active) genes (Figure S3A, top). Reciprocally, we grouped target genes as active or bivalent according to histone modification patterns and compared the expression levels of these two groups. Similarly, bivalent genes (H3K4me3, H3K27me3) in H2A.Z^AP3^ cells showed significant deviations in expression relative to H2A.Z^WT^, whereas active genes did not exhibit significant differences (Figure S3A, bottom, Table S2).

We focused further attention on the class of bivalent genes and compared their expression in H2A.Z^AP3^ relative to H2A.Z^WT^ during ESC differentiation using the Nanostring mCounter assay as an independent measure of gene expression (Figure 3C, Table S4). The subset of genes in the Nanostring probe set included developmental regulators, lineage specific genes as well as pluripotency factors and housekeeping genes, comprising a subset of known H2A.Z target genes and negative controls (Table S4). Consistent with our RNA-Seq data, genes involved in differentiation and pattern specification were expressed at higher levels in H2A.Z^AP3^ ESCs (Figure 3C,D). Interestingly, bivalent genes are expressed at higher levels in H2A.Z^AP3^ relative to H2A.Z^KD^ ESCs, suggesting that incorporation of the mutant results in a distinct chromatin state compared to loss of H2A.Z. Consistent with this idea, H2A.Z^KD^ ESCs failed to activate lineage markers in D3 EBs [10], whereas many of these genes were expressed at higher levels in H2A.Z^AP3^ EBs (Figure 3C,D). The distinct transcriptional output of H2A.Z^AP3^ relative to H2A.Z^KD^ may be due to the replacement of H2A.Z with H2A rather than its loss [36], whereas incorporation of H2A.Z^AP3^ may result in a constitutively dynamic nucleosome.

Given that H2A.Z is enriched at active genes and poised bivalent genes in ESCs (Figure 2A), next we analyzed H3K4me3 and H3K27me3 patterns by ChIP-qPCR in H2A.Z^WT^ and H2A.Z^AP3^ ESCs (Figure 3E,F). While H3K4me3 enrichment patterns did not vary significantly between H2A.Z^WT^ and H2A.Z^AP3^ at either class of H2A.Z target genes (Figure 3E), we found that H3K27me3 enrichment was reduced at bivalent genes in the acidic patch mutant ESCs. This observation is consistent with the de-repression of bivalent genes in H2A.Z^AP3^ ESCs. Together, these results suggest that the divergent H2A.Z residues play key roles in the formation of specialized chromatin domains that are necessary for maintenance of the poised state and for responding to developmental cues.

H2A.Z exists in hybrid nucleosomes along with the histone H3 variant H3.3 whose incorporation marks active promoters, enhancers, and insulator elements [23]–[25]. These double variant nucleosomes are characterized as highly unstable and salt labile, consistent with its enrichment in regions of highly dynamic chromatin. Given that H2A.Z^AP3^ appears to be loosely associated with chromatin relative to H2A.Z^WT^ by salt titration, next we asked whether H3.3 was enriched at bivalent promoters in H2A.Z^AP3^ ESCs compared to H2A.Z^WT^. Since H3.3 differs from H3 by only three amino acids, antibodies against H3.3 cannot be used to effectively distinguish the variant from canonical H3. To circumvent this issue, we transfected an H3.3 C-terminal HA-Flag construct into H2A.Z^WT^ and H2A.Z^AP3^ transgenic ESCs depleted of endogenous H2A.Z (Figure S3B). Similar lines were created with H3.1 C-terminal HA-Flag constructs (Figure S3B). ChIP-qPCR analyses using Flag and HA antibodies revealed that the ratio of H3.3 enrichment relative to H3.1 was higher at target promoters in H2A.Z^AP3^ ESCs relative to H2A.Z^WT^ (Figure 3G). Given that H3.3 is associated with hyperdynamic chromatin, including active genes in ESCs [24]–[26], [47], the increase in H3.3 enrichment at TSSs is consistent with the dynamic nature of H2A.Z^AP3^ nucleosomes and de-repression of target genes in these cells. Taken together, our results demonstrate that the regulation of bivalent genes is highly sensitive to H2A.Z incorporation compared to active genes. In particular, the finding that a subset of genes remained highly expressed in H2A.Z^AP3^ cells during lineage commitment suggests that the acidic patch may be necessary to maintain the poised, silent chromatin state at bivalent genes.

### Divergent acidic patch residues regulate H2A.Z dynamics

**Figure 4:**
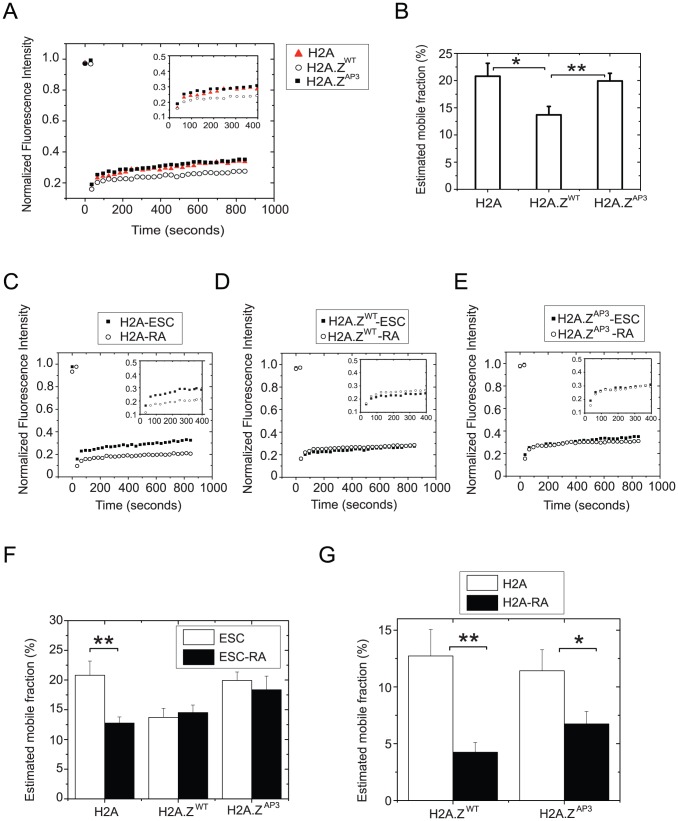
H2A.Z acidic patch influences chromatin stability and H2A.Z dynamics. (A) Mean recovery curves generated from independent FRAP experiments performed on H2A, H2A.Z^WT^ and H2A.Z^AP3^ expressing ESCs. Curves were generated by plotting mean fluorescence intensity of the bleached region measured every 30 secs for a duration of approximately 15 mins. (B) Recovery curves were used to determine percentage mobile fractions. (C–E) Similar recovery curves were generated from FRAP analyses performed on H2A.Z^WT^, H2A, and H2A.Z^AP3^ ESCs subjected to retinoic acid (RA)-induced differentiation for 5 days. (F) Graph representing the estimated mobile fractions of H2A, H2A.Z^WT^ and H2A.Z^AP3^ in ESCs, and RA differentiated cells. *p<0.02, **p<0.004. NS indicates p values >0.6. P values were calculated using the standard unpaired Student t-test. Mean recovery curves were generated from individual curves from n>14 distinct cells for each cell type and condition. (G) Graph representing the estimated mobile fractions of H2A-mCherry in H2A.Z^WT^ and H2A.Z^AP3^ (YFP transgenes) ESCs and day 5 RA differentiated cells. *p<0.04, **p<0.004. P values were calculated using the standard unpaired Student t-test. Mean recovery curves were generated from individual curves from n>12 distinct cells for each cell type and condition.

Nucleosome dynamics have important functional consequences on gene regulation [47]–[49]. Our data suggest that disruption of the divergent residues in the acidic patch results in a more dynamic association of H2A.Z^AP3^ in chromatin. Single cell analysis by fluorescence recovery after photobleaching (FRAP) has been used previously to probe the mobility and dynamics of chromatin-associated proteins in mammalian nuclei [48], [50]–[52]. FRAP studies showed that the mobility of core histones H2B-GFP and H3-YFP are significantly higher in ESCs compared to differentiated cell types [47]. These analyses led to the idea that ESC chromatin is in a hyperdynamic and transcriptionally-permissive state, whereas heterochromatin formation during differentiation leads to a decrease in core histone dynamics [47]. Furthermore, *in vitro* biophysical studies showed that H2A.Z-containing nucleosomal arrays impeded higher order chromatin folding compared to canonical nucleosomes, suggesting that H2A.Z incorporation may contribute to a unique chromatin environment [53]. Thus, we analyzed the recovery kinetics of H2A.Z^WT^-YFP relative to H2A-YFP in ESCs. As expected, H2A-YFP displayed recovery kinetics in ESCs similar to previous reports for H2B-GFP (∼20% mobile fraction) (Figure 4A,B, Table S5) [47]. Interestingly, the dynamics of H2A.Z^WT^ was reduced compared to H2A in ESCs. For example, the mobile fraction of H2A was ∼20% compared to ∼13% for H2A.Z^WT^ (P<0.02) (Figure 4B). Importantly, unbleached photo-imaging controls showed that imaging conditions did not incur inadvertent bleaching and loss of histone fluorescence signal during the experiment (Figure S4A, B). The higher mobility of H2A in ESCs is consistent with a global, transcriptionally permissive chromatin environment in ESC, while the slower recovery of H2A.Z^WT^ is suggestive of a more specialized and distinct H2A.Z chromatin state. Our data are also consistent with prior work indicating that H2A.Z incorporation leads to a more stable nucleosome [21], [54] and that H2A.Z nucleosomes promote formation of 30 nm fibers [53].

Next we probed H2A.Z^WT^ dynamics upon lineage commitment. ESCs were differentiated by addition of retinoic acid (RA) for five days and fluorescence recovery was measured on the final day. Consistent with an increase in chromatin condensation during lineage commitment due to heterochromatin formation, we found that H2A was less dynamic upon RA differentiation, as indicated by the reduction in the mobile fraction (∼20% in ESCs versus ∼12.5% in RA differentiated cells, P<0.02) (Figure 4C, F). In contrast, the recovery rate of H2A.Z^WT^ was similar in ESCs and differentiated cells (Figure 4D, F, Table S5). The inability of H2A.Z to form more highly condensed chromatin structures typical of heterochromatin regions may explain why H2A.Z^WT^ dynamics does not display a further reduction upon ESC differentiation. These data also suggest that H2A.Z dynamics is regulated via a different pathway compared to H2A.

Given that H2A.Z^AP3^ enrichment was lower in ESC chromatin by ChIP-Seq, we investigated its mobility in ESCs. We found that H2A.Z^AP3^ recovers significantly faster than H2A.Z^WT^ and displays recovery kinetics similar to H2A (Figure 4A, B). For example, the H2A.Z^AP3^ mobile fraction was higher in ESCs compared to H2A.Z^WT^ (20% versus 13%; P<0.006) and similar to H2A, suggesting that the divergent acidic patch residues play an important role in regulating H2A.Z dynamics. These observations are consistent with *in vitro* solution studies demonstrating that H2A.Z-containing nucleosome arrays harboring mutations in the acidic patch exhibited chromatin folding kinetics similar to H2A [55]. In contrast, the H2A.Z^AP3^ mobile fraction remained significantly higher upon differentiation compared with H2A, suggesting that H2A.Z^AP3^ nucleosomes are structurally distinct (P<0.044) (Figure 4E, F, Table S5). Taken together, these observations indicate that the reduced occupancy of H2A.Z^AP3^ in ESCs is likely due to its increased dynamics and suggest that the divergent H2A.Z acidic patch is important for formation of specialized chromatin states.

The difference in H2A.Z^AP3^ mobility upon RA differentiation relative to H2A may result from failure of these cells to properly differentiate. To determine if the mobile fraction was reflective of impaired differentiation capacity or purely a result attributable to H2A.Z^AP3^ dynamics, we introduced an H2A-mCherry transgene into H2A.Z^WT^ and H2A.Z^AP3^ ESCs. To determine the mobile fraction of H2A-mCherry in both H2A.Z^WT^ and H2A.Z^AP3^, we performed FRAP on Day 0 and Day 5 RA-differentiated ESCs. We found that the H2A dynamics was similar in H2A.Z^WT^ and H2A.Z^AP3^ ESCs (Figure 4G, Figure S4C, D, Table S5) (12.7% and 11.4% respectively), suggesting that expression of the mutant variant does not significantly alter global H2A dynamics in the undifferentiated state. The relative differences in the percent mobile fraction for H2A-mCherry and H2A-YFP (22% versus 12.7%) is likely due to differences in their respective fluorophore properties [56]. Upon RA differentiation, we found H2A dynamics decreased in H2A.Z^WT^ (4.3% in RA cells), consistent with formation of condensed heterochromatin during lineage commitment (Figure 4G, Figure S4C, Table S5). Similarly H2A dynamics was also reduced in H2A.Z^AP3^ retinoic acid induced cells (6.7% in RA cells), indicating that these cells form chromatin structures more similar to differentiated cell types (Figure 4G, Figure S4D, Table S5). Collectively, these data suggest that the acidic patch region is necessary for proper regulation of H2A.Z dynamics and that its incorporation directly controls gene expression during lineage commitment.

### H2A.Z acidic patch is necessary for transcription-dependent chromatin dynamics

**Figure 5:**
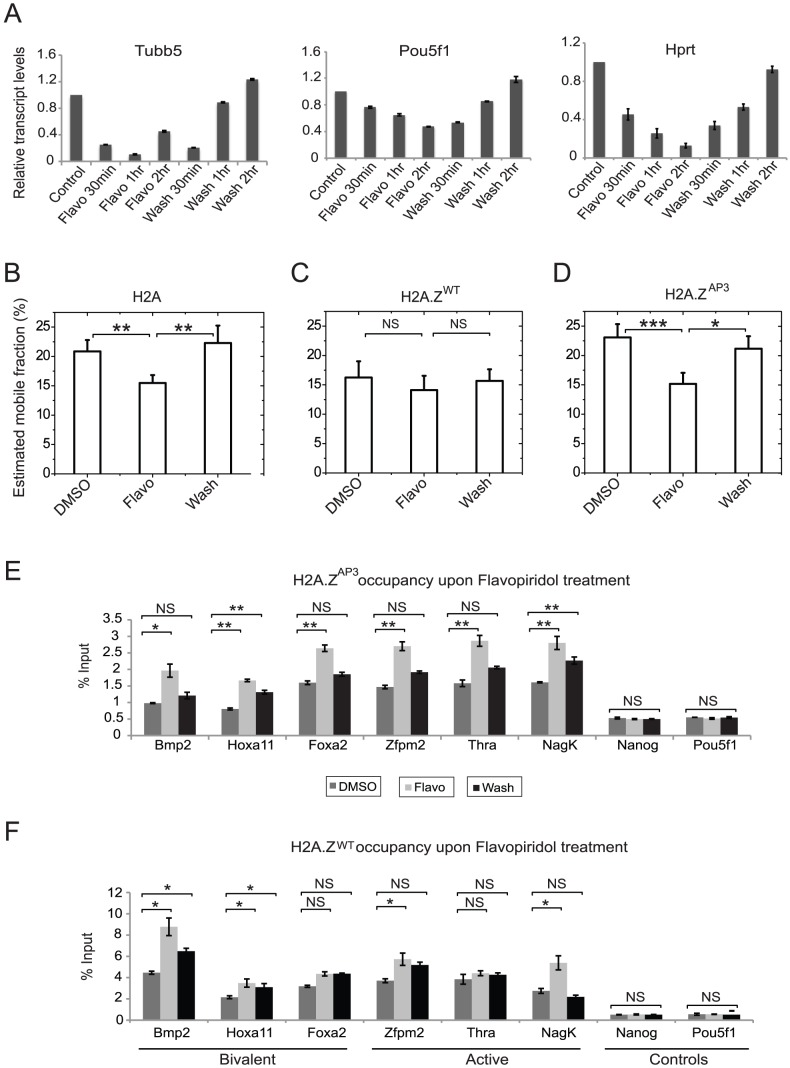
Disruption of H2A.Z acidic patch increases chromatin dynamics in a transcription-dependent manner. (A) qRT-PCR results showing a decrease in the transcript levels of active genes- *Oct4*, *Tubb5*, and *Actin*, upon treatment of 1 µM flavopiridol and restoration of transcript levels upon removal of the agent at the various time points in H2A.Z^WT^ ESCs. (B–D) Graphs representing the estimated mobile fractions in H2A (B), H2A.Z^WT^ (C), and H2A.Z^AP3^ (D) expressing ESCs upon treatment and removal of flavopiridol. ESCs treated with DMSO were used as a control. P values were generated by standard unpaired Student's t-test. *p<0.03, **p<0.015 and NS indicates p values >0.6. Quantitative PCR on ChIP DNA generated by using GFP antibodies in H2A.Z^AP3^ (E) and H2A.Z^WT^ (F) ESCs treated with DMSO, 1 µM flavopiridol (Flavo) and 2 hrs after flavopiridol removal (Wash). Student's t-test was performed to test for statistical significance. * p<0.03, ** p<0.01, and NS represents not significant (>0.05). Error bars represent standard deviations from a triplicate set of experiments.

Active transcription is accompanied by rapid exchange of histone H2A/H2B dimers to accommodate the transiting RNA polymerase [48], [49], [51], [52]. Given that H2A.Z^AP3^ incorporation leads to altered chromatin dynamics and changes in gene expression, we hypothesized that the increase in H2A.Z^AP3^ mobility was linked to transcription. To test this idea, H2A.Z^WT^, H2A.Z^AP3^, and H2A ESCs were treated with flavopiridol, a reversible inhibitor of CDK9 that rapidly decreases RNAP2-dependent transcription. Consistent with previous observations, treatment of H2A.Z^WT^ ESCs with 1 µM flavopiridol for 2 hrs resulted in a 30–44% decrease in RNAP2-dependent transcripts [57], [58] (Figure 5A). Notably, transcript levels were restored to control levels 2 hrs after the wash step. Prior FRAP studies in differentiated cells (HeLa) demonstrated a 3% reduction in the H2B mobile fraction upon treatment with the transcription inhibitor 5,6-Dichlorobenzimidazole 1-β-D-ribofuranoside (DRB) [52]. While this overall fraction is low, it is expected that only a small subset of total H2B is associated with transcription given its broad distribution across the genome. Similarly, we found that H2A treated with flavopiridol exhibited an approximately 6% decrease in the mobile fraction in ESCs by FRAP relative to the DMSO control (P<0.02) (Figure 5B, Figure S5A). Importantly, H2A dynamics were restored to normal levels 2 hrs after removal of flavopiridol, suggesting that at least 6% of H2A in ESCs is linked to transcription (Figure 5B, Table S5). In contrast, we did not detect a measurable change in H2A.Z^WT^ dynamics upon flavopiridol treatment as compared with untreated ESCs, suggesting H2A.Z^WT^ dynamics is not specifically dependent on active transcription (Figure 5C, Figure S5B, Table S5). This result is consistent with the idea that H2A.Z incorporation results in an inherent steady-state dynamic at promoters [21], [54]. It is also possible that ESCs treated with flavopiridol show a moderate reduction in the H2A.Z^WT^ mobile fraction that cannot be effectively resolved by FRAP [59]. In contrast, H2A.Z^AP3^ dynamics was decreased in ESCs upon flavopiridol treatment relative to H2A.Z^WT^ (Figure 5D and Figure S5C). For example, we detected a ∼6% decrease in the H2A.Z^AP3^ mobile fraction upon flavopiridol treatment (P<0.01) similar to H2A, which was restored to near normal levels 2 hrs after flavopiridol removal. These observations were independently confirmed using DRB, an irreversible inhibitor of CDK9 and RNAP2-dependent transcription (Figure S5D–G, Table S5). Thus, our analysis suggests that the altered dynamics observed in H2A.Z^AP3^ ESCs are, in part, linked to transcription. These data are also consistent with disruption of a repressive chromatin state and higher expression of bivalent genes in H2A.Z^AP3^ cells.

To determine if the transcription-dependent decrease in H2A.Z^AP3^ dynamics is coincident with increase in H2A.Z^AP3^ occupancy at target genes, we performed ChIP on H2A.Z^AP3^ ESCs treated with DMSO, flavopiridol (Flavo) and 2 hours post flavopiridol removal (Wash). Consistent with our hypothesis, we found a significant increase in H2A.Z^AP3^ enrichment at target gene promoters with flavopiridol and partial reversal of this trend upon removal of flavopiridol (Figure 5E), suggesting that the decrease in H2A.Z^AP3^ dynamics upon flavopiridol treatment is coincident with higher H2A.Z chromatin occupancy. These data also support our findings that H2A.Z^AP3^ is properly incorporated at promoters. Similar ChIP analyses in H2A.Z^WT^ ESCs revealed a more modest increase in H2A.Z^WT^ occupancy at some but not all target genes upon flavopiridol treatment relative to H2A.Z^AP3^ (Figure 5F). This is also consistent with the FRAP data which indicates minimal change in H2A.Z^WT^ mobile fraction upon flavopiridol treatment. These data are also consistent with *in vitro* biophysical studies showing that an intact H2A.Z acidic patch is necessary for the ability of a nucleosomal template to fold into a 30-nm chromatin fiber and for the efficient repression of transcription [45], [46]. Taken together, our work demonstrates that the divergent acidic patch is an important structural feature that mediates H2A.Z dynamics and maintenance of chromatin states necessary for regulation of inducible gene expression programs during lineage commitment.

## Discussion

**Figure 6:**
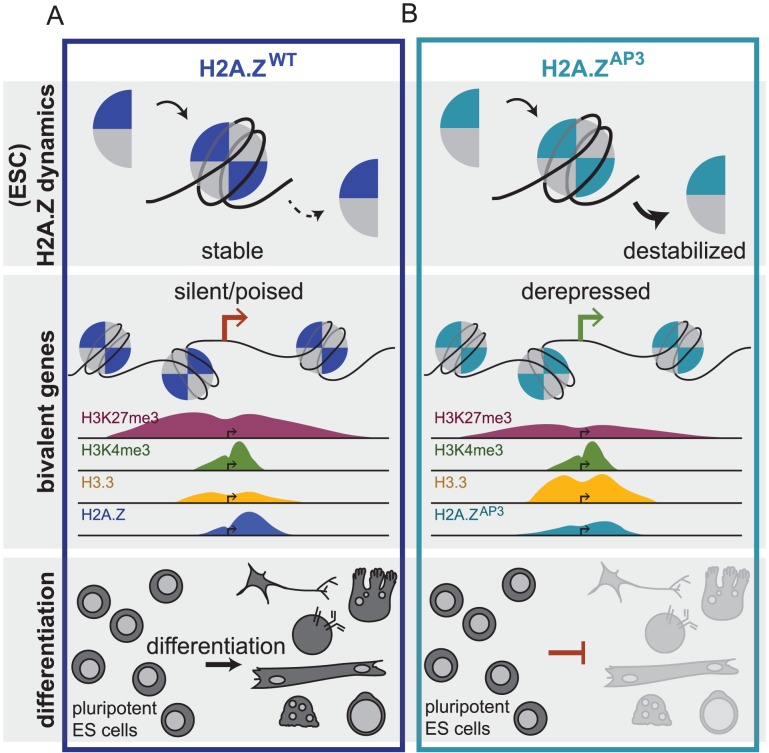
H2A.Z acidic patch couples chromatin dynamics to gene expression regulation during ESC differentiation. Schematic depicting H2A.Z dynamics in H2A.Z^WT^ (A) and H2A.Z^AP3^ (B) ESCs and its consequence on gene expression of developmental genes and ultimately lineage commitment. The black arrow represents similar on rate for H2A.Z -containing dimers in H2A.Z^WT^ and H2A.Z^AP3^ ESCs while a bold black arrow represents a greater off rate for H2A.Z^AP3^-H2B dimers whereas the hashed black arrow represents weaker off rate for H2A.Z-H2B dimers in H2A.Z^WT^. The model demonstrates how the divergent acidic patch domain functions to couple H2A.Z dynamics with the regulation of gene expression programs during ESC differentiation.

The essential histone variant H2A.Z has specialized functions that distinguish it from canonical H2A. The functional distinction between histone variants and core histones is mediated in large part by divergent regions in their respective amino acid sequences. These differences can impact how each histone is incorporated, the stability of the resulting nucleosome, higher order chromatin structure and its interactions with other factors. For example, H2A.Z harbors a unique extended acidic patch that contains three divergent residues compared to H2A. This region of H2A.Z is essential for development in *Drosophila* and *Xenopus* suggesting that it contributes specialized functions [6], [28], [60]. How the acidic patch domain influences H2A.Z function, however, is poorly understood. Here, we find that control of H2A.Z dynamics is mediated through the unique acidic patch region and that this regulation is critical for proper induction of developmental gene expression programs (Figure 6).

### H2A.Z acidic patch is necessary for proper regulation of bivalent genes and formation of specialized chromatin states during ESC differentiation

We analyzed the role of the three divergent H2A.Z acidic patch residues during ESC differentiation. While disruption of the acidic patch domain did not affect the global distribution of H2A.Z, its levels at target gene promoters were reduced, suggesting that the divergent acidic patch domain is an important determinant of H2A.Z incorporation or dynamics. We found that H2A.Z^AP3^ is less stably associated with chromatin relative to H2A.Z^WT^ by salt extraction studies, suggesting that an intact acidic patch is necessary for stabilizing H2A.Z nucleosome structure. The dynamic association of H2A.Z^AP3^ with chromatin is also consistent with its lower enrichment at genomic sites. Prior *in vitro* studies show that H2A.Z forms stable 30 nm fibers at the expense of self-association [53], indicating that the unique acidic patch may be important for mediating distinct higher order chromatin structures.

H2A.Z is found at most H3K4me3 promoters of both active and poised genes and often flanks the nucleosome free region (NFR) around TSSs. We observed a particular reduction in H2A.Z^AP3^ levels in regions corresponding to the nucleosome downstream of the TSS, which likely represents the +1 nucleosome. The +1 nucleosome possesses significant regulatory potential and it is an important mediator of how a gene responds to environmental and developmental cues [39]. For example, a physical interaction between +1 nucleosome and stalled RNAP2 was demonstrated in *Drosophila* S2 cells and studies in *C. elegans* and *Drosophila* suggested that H2A.Z incorporation at the +1 nucleosome is important for maintaining the paused state of promoters [61]–[63]. Consistent with these findings, the accessibility of the TSS is determined in part by the displacement of the +1 nucleosome, which is important for recruitment of RNAP2. Additionally, the +1 nucleosome appears to be important for regulating transcriptional elongation through interactions with Mediator components [40]. Thus, H2A.Z incorporation at the +1 nucleosome may be critical for determining the specific transcriptional response to developmental cues. A similar phenomenon regarding the role of the H2A.Z +1 nucleosome has been observed in plants in response to changes in ambient temperature [15]. Notably, specific transcriptional responses cannot be achieved by replacement with H2A reinforcing the idea that H2A.Z incorporation leads to specialized chromatin states. Similarly, while H2A can substitute for H2A.Z upon loss of the variant in ESCs, target genes fail to activate properly during ESC differentiation [10], [36]. In contrast, incorporation of H2A.Z^AP3^ at TSSs, which is associated with a decrease in the +1 nucleosome, would allow for recruitment of RNAP2 and/or transcriptional elongation consistent with the persistent higher expression of bivalent genes during ESC differentiation (see below). Together, these data point to a critical role for regulating H2A.Z dynamics, particularly at the +1 nucleosome.

The decrease in H2A.Z^AP3^ enrichment led to changes in gene expression particularly at bivalent genes during ESC differentiation. H2A.Z is largely associated with active genes whose promoters are marked by H3K4me3 and it is enriched at a smaller subset of bivalent genes (comprising H3K4me3 and H3K27me3 modifications) in ESCs that are silent yet competent for activation. Why is the regulation of bivalent genes more sensitive to mutations in the acidic patch relative to active genes? We propose that the acidic patch is necessary for mediating H2A.Z dynamics or nucleosome stability upon incorporation at both poised and active target genes. Mutations in the acidic patch increase H2A.Z mobility and possibly alter the stability of the +1 nucleosome, which leads to expression of bivalent genes. Conversely, the increase in H2A.Z dynamics may have little impact on the expression of actively transcribed genes since elongation is occurring at these genes and perhaps other modes of regulation prevail to maintain genes in an active state [48]. This idea is also consistent with the observation that H2A.Z levels decrease upon gene activation [8].

In addition to H2A.Z, Polycomb Repressive Complexes (e.g. PRC1 and PRC2) are enriched at bivalent genes [10], [11]. PRCs are important for establishment and maintenance of repressed chromatin states and play key roles in dynamic regulation of gene expression during lineage commitment [64]. We suggest that disruption of the H2A.Z acidic patch alters the chromatin state and loss of PcG-mediated repression at bivalent genes. Consistent with this idea, we found that H3K27me3 levels were reduced at bivalent genes in H2A.Z^AP3^ ESCs, whereas H3K4me3 levels remained largely unchanged. These data further support our prior findings that functional coordination between PRCs and H2A.Z is important for regulating developmental gene expression patterns during ESC differentiation [10], [65]. Thus, the unique H2A.Z acidic patch may generally restrict the mobility of nucleosomes in ESCs. Changes in local chromatin compaction due to incorporation of the mutant may lead to changes in gene expression and subsequent loss of PRCs leading to a shift in the balance of chromatin associated factors that promote activation. In support of this model, we found that bivalent promoters gained H3.3 in H2A.Z^AP3^ ESCs, a histone variant typically incorporated at highly dynamic chromatin regions. Together, our data suggest that the divergent H2A.Z acidic patch is necessary for the formation of specialized chromatin states that allow for maintenance of the poised state and for proper induction of gene expression programs in response to developmental cues (Figure 6).

### The H2A.Z acidic patch mediates histone dynamics in response to transcriptional cues

Given that H2A.Z displayed reduced chromatin association in ESCs compared to H2A.Z^WT^, we used single cell analysis to further analyze its dynamic association with chromatin. Interestingly, FRAP measurements revealed that H2A.Z was less dynamic than H2A as indicated by its reduced mobile fraction. These data are consistent with the idea that H2A.Z incorporation leads to the formation of a more stable nucleosome and the H2A.Z containing nucleosome arrays promote formation of 30 nm fibers [53]. While FRAP measurements represent overall steady-state chromatin dynamics, little is known regarding the on- and off-rates of H2A.Z. We suggest that this equilibrium is altered upon disruption of the divergent acidic patch residues resulting in a higher off rate and increased transcriptional output at genes that required H2A.Z for maintenance of the poised state (Figure 6). Moreover, our data suggest that the divergent acidic patch is a critical determinant of H2A.Z dynamics and that this region may be important for rapid activation of developmental programs. Remarkably, we show that while H2A.Z^AP3^ appears more dynamic, treatment with the transcriptional inhibitor flavopiridol led to a decrease in its mobile fraction as well as increased occupancy at target promoters. Thus, H2A.Z^AP3^ dynamics may also be regulated by the local chromatin environment as well as by interactions with other factors. Consistent with this idea, studies in Arabidopsis showed that H2A.Z is an important component of the thermosensory response. For example, H2A.Z was less sensitive to nuclease digestion at silent, inducible genes at normal temperature [15]. Upon activation of these genes by increasing temperature, H2A.Z-containing nucleosomes become more accessible to nuclease digestion suggesting that the variant is dynamically remodeled in response to inductive cues. Thus, it remains possible that H2A.Z modifications or interaction with other factors may mediate the function of the divergent acidic patch.

H2A.Z localization and dynamics is regulated from yeast to human in large part by two ATP-dependent remodelers, SRCAP and INO80, [66]–[69]. Thus, future studies will be important to determine how the divergent acidic patch cooperates with chromatin remodelers. For example, INO80 appears to be an important regulator of H2A.Z removal by catalyzing the exchange of H2A.Z for H2A [69]. Thus, one possibility is that H2A.Z^AP3^ is a better substrate for INO80 leading to a higher off rate and increased dynamics. Interestingly, Swc2 in yeast, a conserved subunit of SRCAP, binds directly to H2A.Z [70] and recent work indicates that this interaction may function as a “lock” that prevents rapid exchange of H2A.Z in chromatin [71]. Notably, domain swap experiments in yeast, where the H2A.Z C-terminal domain including the extended acidic patch is replaced with the analogous region of H2A blocks interaction with Swc2 and many other components of the SWR1 remodeling complex [70]. In contrast, deletion of the H2A.Z C-terminal region distal to the extended acidic patch maintained interactions with SWR1 [30]. Consistent with these observations, we found that H2A.Z^AP3^ interacted with SRCAP; however, it is possible that mutations in the extended acidic patch interfere with a specific interaction with YL1, the mammalian Swc2 homologue. Thus, an alternative scenario is that H2A.Z^AP3^ incorporation leads to SRCAP-mediated exchange of H2A.Z for H2A, which would likely influence dynamics.

The acidic patch has been proposed to mediate interactions with histone tails from neighboring nucleosomes, and this domain may also form a novel interaction surface for recruitment of other chromatin factors and downstream effectors [27], [53], [55], [60]. Thus, the unique H2A.Z acidic patch may interact with distinct subsets of factors that determine its specialized functions compared to H2A. In support of this idea, a recent proteomics analysis identified a number of chromatin-associated proteins enriched with H2A.Z [72]. These interactions may be important for regulating transcriptional output at target genes, ultimately allowing the cell to tune signals into specific responses that lead to changes in cell fate. In addition to its divergent structural features, H2A.Z is also subject to numerous posttranslational modifications (PTMs). For example, acetylation of the H2A.Z amino-terminus is a hallmark of active genes and has been implicated in gene regulation and chromosome stability [73]–[76], whereas the carboxy-terminal domain is modified by ubiquitylation and sumolyation, and these marks have been implicated in heterochromatin formation and DNA repair, respectively [77], [78]. As such, PTMs may regulate H2A.Z dynamics by promoting or inhibiting interactions with regulatory factors or by affecting its interactions with neighboring nucleosomes. Overall, our work provides critical insights into the role of the divergent H2A.Z acidic patch residues as a structural determinant that links chromatin dynamics, gene regulation, and ultimately cell fate. Moreover, these data provide a mechanistic explanation for the essential role of the divergent acidic patch during metazoan development.

## Materials and Methods

### Culture of mouse embryonic stem cells (mESCs)

V6.5 (129SvJae and C57BL/6) ESCs were plated with irradiated murine embryonic fibroblasts (MEFs) and grown under typical ESC conditions on gelatinized tissue culture plates. Briefly, cells were grown in Knockout DMEM (Invitrogen) supplemented with 10% fetal bovine serum (Hyclone), leukemia inhibitory factor [16], non-essential amino acids (Invitrogen), L-glutamine (Invitrogen), and penicillin/streptomycin (Invitrogen) as previously described [79]. ESCs used for ChIP-Seq, RNA-Seq and qRT-PCR experiments were plated without MEFs for the final passage.

### Transgenic ESC lines

H2A.Z^WT^-YFP, Mutant H2A.Z^AP3^-YFP and H2A-YFP constructs were generated from the pAd-cDNA (Addgene), which contains a dox-inducible CMV-promoter. The H2A.Z^AP3^ mutant was generated by replacing H2A.Z residues G92, D97, and S98 with the corresponding H2A amino acids (N, N and K respectively) using the instructions provided in the Quikchange Site Directed Mutagenesis Kit (Stratagene). We then replaced GFP in the vector with YFP (YFP exhibits lower background for imaging) to generate an in frame fusion C-terminal to H2A.Z. The resulting lentiviral constructs were transfected into 293 cells using the protocol outlined by the RNAi consortium (BROAD Institute, http://www.broadinstitute.org/rnai/public/). The viral supernatant generated 48 hrs after transfection was used to infect KH2 ESCs [80] to generate wild-type and mutant H2A.Z transgenic ESC lines. The YFP transgenic ESCs were induced with 1 µg/ml of doxycycline and FACS sorted for YFP positive cells.

### shRNA-mediated depletion of endogenous H2A.Z

Lentiviral constructs expressing short hairpins specifically directed at the 3′ UTR of endogenous H2A.Z were introduced into the wild-type and mutant H2A.Z transgenic KH2 ESC lines. Sequences of the different H2A.Z 3′UTR-directed hairpin oligos are as follows: sh#1 5′- AACAGCTGTCCAGTGTTGGTG-3′; sh#2 5′- AATTAGCCTTCCAACCAACCA-3′. Hairpin oligos were annealed and cloned into pLKO.1 vector (Sigma) as detailed by the RNAi consortium, BROAD (http://www.broadinstitute.org/rnai/trc/lib). Since KH2 cells are puromycin resistant [80], blasticidin was used as a selection marker for the generation of endogenous H2A.Z-depleted transgenic KH2 ESCs. The puromycin marker in the pLKO.1 vector was removed by digestion with BamHI and KpnI and replaced with blasticidin. The blasticidin cDNA was PCR amplified from pLenti6.2/V5-DEST Gateway Vector (Invitrogen). V6.5 (129SvJae and C57BL/6) and the YFP transgenic ESCs were cultured as previously described [79]. The endogenous H2A.Z-depleted transgenic KH2 ESCs were cultured in the presence of blasticidin (5 µg/ml) on blasticidin-resistant feeder cells [81].

### ESC differentiation

Retinoic acid-induced differentiation was performed by plating mESCs onto gelatinized tissue culture plates without MEFs and grown in the same mESC media as above but without LIF and supplemented with 1 µM all-trans retinoic acid (Sigma, R2625). Cells were collected after five days. mESCs were induced to form embryoid bodies [68] by plating on non-adherent plates from a starting density of 1–2 million ESCs per 10 ml of mESC media lacking LIF.

### RNA isolation and quantitative real-time PCR

RNA was extracted using TRIzol (Invitrogen, 15596-018) or Izol (5PRIME, 2302700). Purified RNA was reverse transcribed using SuperScript III (Invitrogen, 18080-044) or M-MLV reverse transcriptase (Invitrogen, 28025-013) and random hexamers according to manufacturer protocols. Quantitative PCR reactions were performed with SYBR Green Master Mix (Roche). Primer sequences are listed in Table S4. Relative mRNA levels were determined in triplicate for each transcript using the manufacturer's software (Advanced Relative Quantification with Roche Lightcycler 480 Software Version 1.5) using control genes (*Hprt*, *Gapdh*, or *Tubb5* levels) for normalization.

### RNA-Seq library preparation and analysis methods

RNA was isolated using the protocol mentioned above. The purified RNA was the subjected to oligo (dT) selection, fragmentation and first and double strand synthesis with the Illumina Tru-Seq kit (RS-930-20 01) according to the manufacturer's instructions. DNA fragments above 30 bp was purified using SPRI-TE beads (Beckmann Coulter, Agencourt, A63880) according to manufacturer's instructions. The purified DNA was end-repaired and single A bases for adaptor ligations. The adaptor-ligated DNA was then subjected to double SPRI-TE purification to select for ∼200 bp fragments. These fragments were enriched and barcoded by PCR for multiplexing. A final SPRI-TE purification was performed to clean up the barcoded RNA-Seq libraries.

RNA-Seq data were aligned against the mouse reference genome (mm9) using Tophat 1.4.1 and Bowtie 0.12.7 in single-end mode, tolerating up to 2 mismatches and matches up to 20 locations transcriptome-wide. Gene expression was quantified with Cufflinks 1.3.0, and pairwise differential expression was analyzed using Cuffdiff 1.3.0. H3K4me3 and H3K27me3 enrichment peaks were obtained from our previous work [38]. Histone mark enrichment peaks whose boundaries fell within a region +/−2 kilobases (kbs) of a transcription start sites (TSS, based on RefSeq annotation of the mm9 mouse genome assembly) were identified and the number of H3K4me3 and H3K27me3 TSSs were recorded. RNA-Seq-based isoform expression data from Cufflinks (in RPKMs) were summed over each TSS and used as a metric for the transcriptional output originating from each TSS (Table S2). Median summed isoform expression levels were computed across all isoforms with H3K4me3- or H3K4me3/H3K27me3-marked TSSs in the different ESCs (H2A.Z^WT^, H2A.Z^KD^, and H2A.Z^AP3^) and EB differentiation time points and compared by t-test. Similarly, the overlap between these subsets and the genes differentially regulated upon H2A.Z modulation was tested using the hypergeometric test. Finally, cumulative distribution of isoform expression differences upon H2A.Z modulation were computed by stratifying log2-tranformed isoform fold changes based on the presence of H3K4me3 and H3K27me3-bound regions within +/−2 kbs of the TSS. The significance of the differences between the cumulative distributions plots was assessed using the Kolmogorov-Smirnov test. All the sequencing results in the article have been deposited in GEO under the accession number GSE40065.

### Chromatin Immunoprecipitation (ChIP)

ChIP was performed as described [38] with the following modifications. Diagenode Bioruptor (UCD-200) was used to sonicate with 30 cycles of 30 sec on, 30 sec off. The samples were sonicated in 15 ml polystyrene tubes at 4°C while samples were immersed in ice cold water. Antibodies used for ChIP include: GFP (Abcam, ab290), H2A.Z (Abcam, ab4174), H3K27me3 (Cell Signaling Technology, #9733), H3K4me3 (Millipore, #07-473), Suz12 (Bethyl, A302-407A) and RNAP2 Ser5P (Abcam, ab5131).

### ChIP and site-specific PCR

ChIP enriched DNA was quantified by quantitative PCR and the data analyses performed as described in [10]. qPCR reactions using SYBR Green (Roche) and gene-specific primers (Table S4) were performed on ChIP and whole cell extract (WCE) DNA. Reactions were performed in triplicate on the Roche LightCycler 480. % Input was calculated with the following formula: % Input = 2^(Cp(WCE)-Cp(IP))^×(% WCE).

### ChIP-Seq library preparation and analysis methods

Approximately 200 ng of DNA was submitted to SPRI-works Fragment Library System I (Beckman Coulter) for each library prepared and sequenced on Illumina GAII. ChIP-seq reads were aligned to the mm9 genome assembly using Bowtie 0.12.3, allowing for two mismatches. To determine regions of the genome enriched for H2A.Z, mapped reads were extended to 200 bp (average fragment length) and allocated in 25-bp bins. A Poissonian model was used to determine statistically enriched bins with a P-value threshold set at 1E-9 as described previously [82]. Additionally, we required that genomic bins were at least 5 fold over input to be considered enriched peaks. All the sequencing results in the article have been deposited in GEO under the accession number GSE40065.

### Fluorescence recovery after photobleaching (FRAP) analysis

ESCs were plated in the absence of MEFS on 0.2% gelatin in 2 mm Lab-Tek Chambered #1 borosilicate cover glass chambers containing a 1 mm glass slide 24 hours prior to imaging. Phenol-red free DMEM (Invitrogen 21063-045) was used to make ESC media for imaging purposes to minimize medium auto fluorescence. For differentiation experiments, ESCs were treated with 1 µM retinoic acid and plated on 0.2% gelatin in Lab-Tek Chambered cover glass 24 hours prior to imaging. YFP transgenic cells were additionally induced with 1 µg/ml of doxycycline 24 hours prior to imaging. Confocal fluorescence imaging was performed on a LSM 510 microscope (Carl Zeiss, Jena, Germany) with a 514 nm wavelength laser for YFP excitation, a 520–550 nm bandpass emission filter and a 100× 1.4 N. A. oil immersion objective. FRAP experiments were performed by photobleaching for a short 5.4 sec exposure to 100% laser intensity. To minimize error in the quantification of fluorescence recovery induced by the movement of unbleached chromatin into the bleached region due to the dynamic nature of ESC chromatin and changes in nuclear morphology, a 20 pixel sub-region within the 75 pixel bleached region was used was used to quantify fluorescence recovery using custom-written MATLAB routines (Mathworks, Natick, MA). In all cases the bleached region was sufficiently smaller than the size of the nucleus to minimize the effect of the original loss of fluorescent protein due to bleaching on maximal recovery. In any case, such loss would result in a uniform underestimation of mobile fractions in all tested instances, and not affect relative differences. Images were acquired using 30 sec acquisition intervals for approximately 14 mins with scan speed and imaging intervals optimized to minimize photobleaching during the recovery process. A minimum of 14 cells were analyzed for each FRAP experiment, with background correction performed by normalizing intensities to the maximum initial mean intensity in the bleach spot prior to photobleaching to generate individual FRAP curves. Mean data shown in results constitute the average of individual recovery curves. Mobile fractions (MF) were calculated from individual curves using MF = (I_dip_−I_sat_)/(1−I_dip_), where I_dip_ is the value of the mean intensity immediately after the bleaching pulse, and I_sat_ is the mean intensity at the end of the monitored recovery period. Student's t-test was used to calculate the statistical significance of the differences between estimated mobile fractions. For the flavopiridol experiments, cells were treated with 1 µM flavopiridol 2 hours before conducting photobleaching experiments. The post-wash FRAP experiments were performed on ESCs 2 hrs after washing away the inhibitor. For DRB experiments, ESCs were treated with 15 µM DRB for 2 hours and then imaged for FRAP as described above. All mobile fractions and the respective standard errors are listed in Table S5.

### Histone extraction

Histone extracts were prepared by harvesting the cells first with ice-cold PBS supplemented with 5 mM sodium butyrate (10^6^–10^7^ cells). The cells were then washed in cold filter sterilized Lysis buffer (0.25M Sucrose, 3 mM CaCl_2_, 1 mM Tris pH 8.0, 0.5% NP-40). The nuclei were spun down at 3900 rpm for 5 min at 4°C. The supernatant was removed leaving the pellet of 50 µl of nuclei. This pellet was washed again with Wash solution (300 mM NaCl, 5 mM MgCl_2_, 5 mM DTT, 0.5% NP40) and spun under the same conditions as mentioned above. The supernatant was removed and the nuclei were resuspended in 50 µl of Extraction solution (0.5M HCl, 10% glycerol, 0.1M 2-mercaptoethylamine-HCl). The nuclei were then left in ice for 30 min after which they were spun at 13,000 rpm for 5 min at 4°C. The supernatant was transferred to siliconized tubes and 500 µl of acetone was added. The resulting solution was incubated at −20°C overnight. The resulting histone precipitate was spun at 13,000 rpm for 5 min for further analyses. Rabbit anti-H2A.Z antibody (Abcam, ab4174) and Rabbit anti-GFP antibody (Abcam, ab290) was used for western blot at concentrations recommended by the manufacturer.

### Immunoblot analysis

Histone extracts, lysates and co-immunoprecipitated samples were resolved on SDS-PAGE gels and transferred on PVDF membrane (Bio-rad, 162-0177) using the Mini-Trans-Blot (Bio-rad, 170-3930). The transferred blot is then blocked with 5% milk in TBST (0.1% Tween-20 in 1× Tris-buffered saline-pH 7.4) for 1 hour at room temperature and incubated with the indicated antibodies prepared in TBST with 5% milk. The blots were then washed with TBST three times and incubated with species-specific HRP-conjugated secondary antibodies (Calbiochem). After incubation with secondary antibodies, the immunoblots was washed again with TBST and visualized using HRP substrate from Biorad (170-5070).

### Immunostaining and histology

Cells were fixed with 4% paraformaldehyde for 20 minutes at room temperature, washed 3× with PBS, permeabilized in PBS with 0.2% TritonX, 0.1% Tween-20 for 30 minutes at room temperature, washed twice with PBS and blocked with PBS containing 0.1% Tween-20, 2% CCS (Cosmic Calf Serum, Invitrogen) for 1 hour at room temperature. The cells were then stained with anti-Oct4 antibody (mouse monoclonal, Santa Cruz, sc-5279, 1∶100) for 1 hour at room temperature, then washed twice more with block and stained with anti-mouse secondary (Alexafluor 594). After washing twice more, the cells were imaged using Nikon Eclipse Ti-S.

For histology, the EBs were fixed in 10% formalin for 20–30 mins and washed with PBS. Fixed EBs were then dehydrated by treating with a gradient of 70%, 80%, 90% and 100% ethanol for 20 mins each. The dehydrated EBs were finally cleared in xylene and embedded in paraffin overnight at 60°C. Sections of EBs at 0.4 microns were generated and placed on slides for staining. The sections are deparaffinized with xylene, rehydrated and stained with Harris Hematoxylin (Surgipath, 01560) and Eosin (Polyscientific, s176).

### Qualitative and quantitative (SILAC) proteomics analyses

ESCs were cultured in ^13^C_6_/^15^N_2_-lysine(+8) ^13^C_6_/^15^N_4_-arginine(+10) (“SILAC heavy”) or naturally occurring lysine and arginine (“SILAC light”) medium according to [83]. Histones were purified from ES and differentiated cells essentially as described [84] except that a Zorbax C8 HPLC column was employed (Agilent). Each 1 min fraction collected from the HPLC separation of the histones was subjected to SDS-PAGE. Subsequent LC-MS/MS experiments were performed on an LTQ Velos-Orbitrap mass spectrometer (ThermoFisher Scientific) fed by an Agilent 1200 nano-HPLC system (Agilent) following procedures analogous to those described elsewhere [85]. First, the Coomassie-stained visible bands on PAGE separations of the HPLC fractions were interrogated by tryptic and chymotryptic digestion. Peptides unique to H2A.Z (and not derived from other H2A variants) were detected in bands of ∼14 kDa and ∼20 kDa. The ∼14 kDa variant of H2A.Z co-HPLC-separated with Histone H4 (∼12 kDa) while the ∼20 kDa variant co-HPLC-separated with Histone H2B (∼14 kDa). Next, these bands (from a parallel preparation) were subject to in-gel propionylation using propionic anhydride according to [86]. To study the C-terminal ubiquitination of H2A.Z, chymotryptic peptides were analyzed. To study the N-terminal acetylation of H2A.Z, tryptic peptides were analyzed. *M/z* values corresponding to the various acetylated and ubiquitin-residual peptides (The proteases will cleave ubiquitin as well as H2A.Z, leaving a branched peptide residual) were calculated. Separate acquisition methods were designed for the study of acetylation or ubiquitination. Selective-ion monitoring (SIM) windows were designed around these *m/z* as appropriate and data-independent MS/MS scans were acquired at these *m/z* ratios as dictated by each experiment. The sample was introduced to the mass spectrometer via liquid chromatography with conditions identical to those described [87].

### Cell cycle and apoptosis analyses

#### Cell cycle kinetics using PI

A total of 5 million ESCs were resuspended in ice cold PBS to single-cell suspension and fixed using 95% ethanol at 4°C overnight. The fixed cells were then resuspended in PBS containing 1% BSA containing 50 µg/ml of propidium iodide (PI). The cells were then treated with RNAse A at a final concentration of 1 mg/ml at 37°C for 30 mins. The cells were then resuspended in PBS. DNA peak profiles were generated on a FACSCalibur (Becton Dickinson) flow cytometer at the Swanson Biotechnology Center Flow Cytometry Facility at the Koch Institute-MIT.

#### Cell cycle kinetics using BrdU incorporation

A total of 2 million cells were used for incorporation of BrdU using the BD Pharmingen APC BrdU Flow Kit (Cat.No: 552598) instructions. The ESCs were incubated with a final concentration of 10 µM BrdU for an hour. Day 5 RA-induced ESCs were incubated with the same concentration of BrdU for 3 hrs.

#### Annexin V staining

5 million cells were used for staining with Cy5 Annexin V using the BioVision Annexin V-Cy5 Apoptosis Kit (K103-25) according to manufacturer's instructions.

#### Cells counted from Day 10 embryoid bodies (EBs)

The Day 10 EBs are collected and washed with PBS. The washed EBs were resuspended in trypsin for 5 mins. The trysin was inactivated with ESC media containing serum. The cells were then counted using the Nexcelom Cell counter (Cellometer Auto T4).

### Chromatin spreads

Chromosome spread analysis was performed as described in [88]. The cells were treated with a final concentration of 1 µg/ml of Nocodazole for 8 hours at 37°C. The treated cells were then washed with PBS. The mitosis-arrested cells were then collected and spread on slides using a Cytospin 4 (Thermo, Waltham, MA) [89]. The spread cells were then fixed with paraformaldehyde and permeabilized with detergent. The fixed slides were then stained for CENP-A at a dilution of 1∶800 (Cell Signaling Technology, #2048) and visualized with Alexa 594 secondary antibodies (1∶500). The DNA was visualized using DAPI and the GFP signal was from the YFP-fused transgene expression.

## Supporting Information

Figure S1 The H2AFZ (H2A.Z) isoform is more abundant than the H2AFV isoform. (A) Protein sequence alignment of mouse H2AFZ (H2A.Z), H2AFV and H2A. The teal box surrounds the sequence corresponding to the acidic patch domain and metal-ion binding site, while the red box highlights the amino acids that differ between H2AFZ and H2AFV. (B) Relative protein abundance of H2AFZ and H2AFV in ESCs estimated by SILAC mass spectrometric analyses. (C) Representative real-time PCR analyses showing endogenous H2A.Z mRNA levels (top) and H2AFV transcript levels (bottom) in ESCs expressing H2A.Z YFP fusion transgenes in the presence of two different H2A.Z 3′UTR-specific shRNA sh#1 and sh#2. All the qPCR data is normalized to relative to levels of Tubb5. Standard deviations were determined from three biological replicates. (D) Western blot using H2A.Z antibodies on histone extracts isolated from dox-induced (+) and uninduced (−) H2A.Z^WT^ and H2A.Z^AP3^ transgenic ESC lines in the presence (+) and absence (−) of the H2A.Z 3′UTR-specific shRNA. H3 levels were used as a load control. (E) Table summarizing results from PI analyses performed on unmodified, H2A.Z^KD^, H2A.Z^WT^ and H2A.Z^AP3^. (F) BrdU incorporation analyses were performed to determine cell cycle kinetics in unmodified, H2A.Z^KD^, H2A.Z^WT^ and H2A.Z^AP3^ ESCs. Graph representing the percentage of cells at various stages of the cell cycle in the above mentioned cell lines shows similar distribution of cells, suggesting that H2A.Z-depletion (H2A.Z^KD^) and H2A.Z^AP3^ expression does not impact cell cycle kinetics in ESCs relative to H2A.Z^WT^. (G) BrdU incorporation analyses performed in Day 5 RA-induced differentiation of ESCs reveals similar distribution of cells at various stages of the cell cycle, suggesting that expression of the H2A.Z^AP3^ mutant does not alter cell cycle kinetics upon differentiation. Annexin V Cy5 staining of ES reveals no significant difference in the rate of apoptosis between H2A.Z^WT^, H2A.Z^KD^ and H2A.Z^AP3^ in ESCs (H) and day 10 differentiated EBs (I), suggesting that the expression of H2A.Z^AP3^ mutant does not induce apoptosis in ESCs or upon differentiation. (J) Graph representing the number of cells isolated from H2A.Z^WT^, H2A.Z^KD^ and H2A.Z^AP3^ day 10 EBs.
(EPS)

Figure S2 H2A.Z acidic patch mutant is capable of chromatin incorporation. (A) Real-time PCR showing comparable transcript levels of H2A.Z^WT^ and H2A.Z^AP3^ mutant transgene in the presence of endogenous H2A.Z. (B) Representative western blot showing the enrichment of nuclear, chromatin, and cytoplasmic fractions isolated from H2A.Z^WT^, H2A.Z^AP3^, and uninduced (No DOX) ESCs. GFP antibodies were used to probe for the presence of H2A.Z^WT^ and H2A.Z^AP3^ transgenes in the various cellular fractions. H3 antibodies were used as a marker for nuclear and chromatin fractions and GAPDH was used as a cytoplasmic marker. (C) Chromosome spreads generated from nuclei isolated from H2A.Z^WT^, H2A.Z^AP3^ and H2A stained with DAPI (blue) and centromeric H3 variant-CENP-A (red) suggest that H2A.Z^AP3^ expression shows a similar distribution pattern as H2A.Z^WT^, indicating the ability of H2A.Z^AP3^ to effectively incorporate into DNA in chromosomes. The green fluorescence signal is from the YFP-fused transgene expression (as indicated above each image). Quantitative PCR on ChIP DNA generated using GFP (D) and H3K9me3 (E) antibodies in H2A.Z^WT^ (WT) and H2A.Z^AP3^ (AP3) ESCs and Day 5 RA-differentiated. LINE-L1-1 and 2 refers to primers directed against ORF1 and ORF2 respectively of the LINE-L1 repetitive elements. (F) Co-immunoprecipitation analyses with GFP reveals by western blot that both H2A.Z^WT^ and H2A.Z^AP3^ interact with H2A.Z-specific incorporation module- SRCAP, ATP-dependent DNA helicase RUVBL1 (a subunit of chromatin remodeling complexes INO80, Tip60-p400 and SRCAP) and histone chaperone Nap1, suggesting that the reduced enrichment of H2A.Z^AP3^ genome-wide is not due to its inability to associate with its incorporation complex. (G) ChIP qPCR on DNA immuno-precipitated with H2A.Z antibodies (Abcam, ab4174) in cells over expressing H2A.Z^AP3^ (for more than four passages), in the presence of endogenous H2A.Z (H2A.Z^AP3^/^WT^) show normal H2A.Z localization compared to reduced enrichment of H2A.Z^AP3^ expressing ESCs in an H2A.Z-depleted background (H2A.Z^AP3^/KD), suggesting that H2A.Z^AP3^ interaction with deposition module-SRCAP does not result in an inactive complex. (H) Co-immunoprecipitation analysis with GFP in H2A, H2A.Z^WT^ and H2A.Z^AP3^ ESCs reveals that NAP1 interaction with H2A is weaker that H2A.Z^WT^ and H2A.Z^AP3^. Titrations of 1 µl (white rectangle) and 5 µl (dark rectangle) of the dox-induced immunoprecipitated material were performed to prevent saturation of the chemiluminescent signal thus allowing for the optimal quantification. (I) Graph showing the ratio of NAP1L1 signal to input signal from co-immunoprecipitation analyses performed in H2A, H2A.Z^WT^ and H2A.Z^AP3^ ESCs (in the endogenous H2A.Z knockdown background) (Figure S2H). * p value <0.03, ** p value <0.01. The respective uninduced cells (No DOX) were used as negative controls for above co-immunoprecipitation analyses. All densitometric measurements of the western blots were performed in ImageJ 1.43r. The standard deviations were generated from triplicates.
(TIF)

Figure S3 H2A.Z acidic patch is necessary for precise regulation of developmental genes during ESC lineage commitment. (A) Venn diagrams representing overlap between differentially regulated genes (>1.5 fold) in H2A.Z^AP3^ relative to H2A.Z^WT^ (green) and presence of active (H3K4me3) chromatin mark within +/−2 kb around the TSS (pale pink) at D0 and D3. P-values were calculated by hypergeometric test (Top). Cumulative distribution function plots of fold change for all genes (black), genes with H3K4me3+ only (blue), bivalent (H3K27me+ and H3K4me3+) domains (red) using RNA-Seq data generated from H2A.Z^AP3^ expressing D0 (left) and D3 (right) EBs (bottom). P-values based on the Kolmogorov-Smirnov test statistics of the difference between cumulative distributions of log-transformed expression fold changes between the different TSS groups are indicated for each plot. (B) Western blot analyses of histone extracts generated from H2A.Z^WT^, H2A.Z^KD^ and H2A.Z^AP3^ ESCs containing C terminal Flag-HA tagged H3.1 and H3.3 respectively. H2A was used as load control for this analyses.
(EPS)

Figure S4 FRAP analyses reveals unique role of H2A.Z acidic patch in regulating H2A.Z dynamics during ESC differentiation. (A) Time series of fluorescence images during FRAP analyses in H2A.Z^WT^ ESCs and Day 5 retinoic acid differentiated cells. For analysis of FRAP images, a square region in cells expressing fluorescent fusion protein is bleached and a smaller circle within the bleached regions is used to measure the recovery in fluorescence intensity over ∼15 mins. This enables effective measurement of the fluorescence recovery in the event of large-scale movement of nuclei during the imaging process. (B) Relative intensity plot of unbleached H2A ESCs at various time points during imaging. This analysis includes an average of 10 cells and is normalized relative to the first time-point. Mean recovery curves generated for H2A-mCherry in H2A.Z^WT^ (C) and H2A.Z^AP3^ (D) ESCs and Day 5 of RA-differentiated ESCs from individual recovery curves generated from least 12 cells for each cell type and condition.
(EPS)

Figure S5 Disruption of H2A.Z acidic patch increases chromatin dynamics in a transcription-dependent manner. Mean recovery curves generated for H2A (A), H2A.Z^WT^ (B), and H2A.Z^AP3^ (C) upon treatment and removal of 1 µM flavopiridol from individual curves of n>14 cells for each cell type and condition. DMSO-treatment was used as a control. (D) qRT-PCR analyses showing reduced transcript levels of active genes-*Tubb5*, *Pou5f1*, *Hprt* and *Actin* upon treatment with 15 µM DRB for 2 hours in H2A.Z^WT^ ESCs. Estimated mobile fractions were determined and plotted for H2A (E), H2A.Z^WT^ (F), and H2A.Z^AP3^ (G) upon treatment with DMSO and DRB (5,6-Dichlorobenzimidazole 1-β-D-ribofuranoside) from individual recovery curves generated from at least 13 different cells for each cell type and condition. * p value of <0.018 and NS indicates non-significant p value (>0.35).
(EPS)

Table S1 ChIP-Seq analyses of H2A.Z^WT^ and H2A.Z^AP3^ ESCs.
(XLSX)

Table S2 RNA-Seq analyses in H2A.Z^WT^, H2A.Z^KD^ and H2A.Z^AP3^ during ESC differentiation.
(XLSX)

Table S3 Nanostring analyses of mRNA abundance in H2A.Z^WT^, H2A.Z^KD^ and H2A.Z^AP3^ during ESC differentiation.
(XLSX)

Table S4 PCR primers used for qRT-PCR and ChIP-qPCR analyses.
(XLSX)

Table S5 Mobile fractions calculated from FRAP analyses performed in the different cell lines and conditions used in this study.
(XLSX)

## References

[1] Banaszynski LA, Allis CD, Lewis PW (2010) Histone variants in metazoan development. Dev Cell 19: 662–674. 21074717 10.1016/j.devcel.2010.10.014 PMC3033583

[2] Henikoff S, Ahmad K (2005) Assembly of variant histones into chromatin. Annu Rev Cell Dev Biol 21: 133–153. 16212490 10.1146/annurev.cellbio.21.012704.133518

[3] Luger K, Dechassa ML, Tremethick DJ (2012) New insights into nucleosome and chromatin structure: an ordered state or a disordered affair? Nat Rev Mol Cell Biol 13: 436–447. 22722606 10.1038/nrm3382 PMC3408961

[4] Guillemette B, Gaudreau L (2006) Reuniting the contrasting functions of H2A.Z. Biochem Cell Biol 84: 528–535. 16936825 10.1139/o06-077

[5] Faast R, Thonglairoam V, Schulz TC, Beall J, Wells JR, et al (2001) Histone variant H2A.Z is required for early mammalian development. Curr Biol 11: 1183–1187. 11516949 10.1016/s0960-9822(01)00329-3

[6] Ridgway P, Brown KD, Rangasamy D, Svensson U, Tremethick DJ (2004) Unique residues on the H2A.Z containing nucleosome surface are important for Xenopus laevis development. J Biol Chem 279: 43815–43820. 15299007 10.1074/jbc.M408409200

[7] Draker R, Cheung P (2009) Transcriptional and epigenetic functions of histone variant H2A.Z. Biochem Cell Biol 87: 19–25. 19234520 10.1139/O08-117

[8] Guillemette B, Bataille AR, Gevry N, Adam M, Blanchette M, et al (2005) Variant histone H2A.Z is globally localized to the promoters of inactive yeast genes and regulates nucleosome positioning. PLoS Biol 3: e384. 16248679 10.1371/journal.pbio.0030384 PMC1275524

[9] Sarma K, Reinberg D (2005) Histone variants meet their match. Nat Rev Mol Cell Biol 6: 139–149. 15688000 10.1038/nrm1567

[10] Creyghton MP, Markoulaki S, Levine SS, Hanna J, Lodato MA, et al (2008) H2AZ is enriched at polycomb complex target genes in ES cells and is necessary for lineage commitment. Cell 135: 649–661. 18992931 10.1016/j.cell.2008.09.056 PMC2853257

[11] Ku M, Jaffe JD, Koche RP, Rheinbay E, Endoh M, et al (2012) H2A.Z landscapes and dual modifications in pluripotent and multipotent stem cells underlie complex genome regulatory functions. Genome Biol 13: R85. 23034477 10.1186/gb-2012-13-10-r85 PMC3491413

[12] Raisner RM, Hartley PD, Meneghini MD, Bao MZ, Liu CL, et al (2005) Histone variant H2A.Z marks the 5′ ends of both active and inactive genes in euchromatin. Cell 123: 233–248. 16239142 10.1016/j.cell.2005.10.002 PMC2039754

[13] Azuara V, Perry P, Sauer S, Spivakov M, Jorgensen HF, et al (2006) Chromatin signatures of pluripotent cell lines. Nat Cell Biol 8: 532–538. 16570078 10.1038/ncb1403

[14] Bernstein BE, Mikkelsen TS, Xie X, Kamal M, Huebert DJ, et al (2006) A bivalent chromatin structure marks key developmental genes in embryonic stem cells. Cell 125: 315–326. 16630819 10.1016/j.cell.2006.02.041

[15] Kumar SV, Wigge PA (2010) H2A.Z-containing nucleosomes mediate the thermosensory response in Arabidopsis. Cell 140: 136–147. 20079334 10.1016/j.cell.2009.11.006

[16] Sutcliffe EL, Parish IA, He YQ, Juelich T, Tierney ML, et al (2009) Dynamic histone variant exchange accompanies gene induction in T cells. Mol Cell Biol 29: 1972–1986. 19158270 10.1128/MCB.01590-08 PMC2655607

[17] Hardy S, Jacques PE, Gevry N, Forest A, Fortin ME, et al (2009) The euchromatic and heterochromatic landscapes are shaped by antagonizing effects of transcription on H2A.Z deposition. PLoS Genet 5: e1000687. 19834540 10.1371/journal.pgen.1000687 PMC2754525

[18] Cui K, Zang C, Roh TY, Schones DE, Childs RW, et al (2009) Chromatin signatures in multipotent human hematopoietic stem cells indicate the fate of bivalent genes during differentiation. Cell Stem Cell 4: 80–93. 19128795 10.1016/j.stem.2008.11.011 PMC2785912

[19] Bernstein BE, Kamal M, Lindblad-Toh K, Bekiranov S, Bailey DK, et al (2005) Genomic maps and comparative analysis of histone modifications in human and mouse. Cell 120: 169–181. 15680324 10.1016/j.cell.2005.01.001

[20] Abbott DW, Ivanova VS, Wang X, Bonner WM, Ausio J (2001) Characterization of the stability and folding of H2A.Z chromatin particles: implications for transcriptional activation. J Biol Chem 276: 41945–41949. 11551971 10.1074/jbc.M108217200

[21] Park YJ, Dyer PN, Tremethick DJ, Luger K (2004) A new fluorescence resonance energy transfer approach demonstrates that the histone variant H2AZ stabilizes the histone octamer within the nucleosome. J Biol Chem 279: 24274–24282. 15020582 10.1074/jbc.M313152200

[22] Park YJ, Luger K (2008) Histone chaperones in nucleosome eviction and histone exchange. Curr Opin Struct Biol 18: 282–289. 18534842 10.1016/j.sbi.2008.04.003 PMC2525571

[23] Goldberg AD, Banaszynski LA, Noh KM, Lewis PW, Elsaesser SJ, et al (2010) Distinct factors control histone variant H3.3 localization at specific genomic regions. Cell 140: 678–691. 20211137 10.1016/j.cell.2010.01.003 PMC2885838

[24] Jin C, Zang C, Wei G, Cui K, Peng W, et al (2009) H3.3/H2A.Z double variant-containing nucleosomes mark ‘nucleosome-free regions’ of active promoters and other regulatory regions. Nat Genet 41: 941–945. 19633671 10.1038/ng.409 PMC3125718

[25] Jin C, Felsenfeld G (2007) Nucleosome stability mediated by histone variants H3.3 and H2A.Z. Genes Dev 21: 1519–1529. 17575053 10.1101/gad.1547707 PMC1891429

[26] Thakar A, Gupta P, Ishibashi T, Finn R, Silva-Moreno B, et al (2009) H2A.Z and H3.3 histone variants affect nucleosome structure: biochemical and biophysical studies. Biochemistry 48: 10852–10857. 19856965 10.1021/bi901129e

[27] Suto RK, Clarkson MJ, Tremethick DJ, Luger K (2000) Crystal structure of a nucleosome core particle containing the variant histone H2A.Z. Nat Struct Biol 7: 1121–1124. 11101893 10.1038/81971

[28] Clarkson MJ, Wells JR, Gibson F, Saint R, Tremethick DJ (1999) Regions of variant histone His2AvD required for Drosophila development. Nature 399: 694–697. 10385122 10.1038/21436

[29] Jensen K, Santisteban MS, Urekar C, Smith MM Histone H2A.Z acid patch residues required for deposition and function. Mol Genet Genomics 285: 287–296. 21359583 10.1007/s00438-011-0604-5 PMC3253533

[30] Wang AY, Aristizabal MJ, Ryan C, Krogan NJ, Kobor MS (2011) Key functional regions in the histone variant H2A.Z C-terminal docking domain. Mol Cell Biol 31: 3871–3884. 21791612 10.1128/MCB.05182-11 PMC3165728

[31] Wratting D, Thistlethwaite A, Harris M, Zeef LA, Millar CB A conserved function for the H2A.Z C terminus. J Biol Chem 287: 19148–19157. 22493515 10.1074/jbc.M111.317990 PMC3365947

[32] Jaenisch R, Young R (2008) Stem cells, the molecular circuitry of pluripotency and nuclear reprogramming. Cell 132: 567–582. 18295576 10.1016/j.cell.2008.01.015 PMC4142810

[33] Murry CE, Keller G (2008) Differentiation of embryonic stem cells to clinically relevant populations: lessons from embryonic development. Cell 132: 661–680. 18295582 10.1016/j.cell.2008.02.008

[34] Dryhurst D, Ishibashi T, Rose KL, Eirin-Lopez JM, McDonald D, et al (2009) Characterization of the histone H2A.Z-1 and H2A.Z-2 isoforms in vertebrates. BMC Biol 7: 86. 20003410 10.1186/1741-7007-7-86 PMC2805615

[35] Eirin-Lopez JM, Gonzalez-Romero R, Dryhurst D, Ishibashi T, Ausio J (2009) The evolutionary differentiation of two histone H2A.Z variants in chordates (H2A.Z-1 and H2A.Z-2) is mediated by a stepwise mutation process that affects three amino acid residues. BMC Evol Biol 9: 31. 19193230 10.1186/1471-2148-9-31 PMC2644675

[36] Hu G, Cui K, Northrup D, Liu C, Wang C, et al (2013) H2A.Z facilitates access of active and repressive complexes to chromatin in embryonic stem cell self-renewal and differentiation. Cell Stem Cell 12: 180–192. 23260488 10.1016/j.stem.2012.11.003 PMC3570599

[37] Barski A, Cuddapah S, Cui K, Roh TY, Schones DE, et al (2007) High-resolution profiling of histone methylations in the human genome. Cell 129: 823–837. 17512414 10.1016/j.cell.2007.05.009

[38] Wamstad JA, Alexander JM, Truty RM, Shrikumar A, Li F, et al (2012) Dynamic and coordinated epigenetic regulation of developmental transitions in the cardiac lineage. Cell 151: 206–220. 22981692 10.1016/j.cell.2012.07.035 PMC3462286

[39] Jiang C, Pugh BF (2009) Nucleosome positioning and gene regulation: advances through genomics. Nat Rev Genet 10: 161–172. 19204718 10.1038/nrg2522 PMC4860946

[40] Nock A, Ascano JM, Barrero MJ, Malik S (2012) Mediator-regulated transcription through the +1 nucleosome. Mol Cell 48: 837–848. 23159738 10.1016/j.molcel.2012.10.009 PMC3534924

[41] Nekrasov M, Amrichova J, Parker BJ, Soboleva TA, Jack C, et al (2012) Histone H2A.Z inheritance during the cell cycle and its impact on promoter organization and dynamics. Nat Struct Mol Biol 19: 1076–1083. 23085713 10.1038/nsmb.2424

[42] Ruhl DD, Jin J, Cai Y, Swanson S, Florens L, et al (2006) Purification of a human SRCAP complex that remodels chromatin by incorporating the histone variant H2A.Z into nucleosomes. Biochemistry 45: 5671–5677. 16634648 10.1021/bi060043d

[43] Wong MM, Cox LK, Chrivia JC (2007) The chromatin remodeling protein, SRCAP, is critical for deposition of the histone variant H2A.Z at promoters. J Biol Chem 282: 26132–26139. 17617668 10.1074/jbc.M703418200

[44] Luk E, Vu ND, Patteson K, Mizuguchi G, Wu WH, et al (2007) Chz1, a nuclear chaperone for histone H2AZ. Mol Cell 25: 357–368. 17289584 10.1016/j.molcel.2006.12.015

[45] Thakar A, Gupta P, McAllister WT, Zlatanova J (2010) Histone variant H2A.Z inhibits transcription in reconstituted nucleosomes. Biochemistry 49: 4018–4026. 20387858 10.1021/bi1001618

[46] Zhou J, Fan JY, Rangasamy D, Tremethick DJ (2007) The nucleosome surface regulates chromatin compaction and couples it with transcriptional repression. Nat Struct Mol Biol 14: 1070–1076. 17965724 10.1038/nsmb1323

[47] Meshorer E, Yellajoshula D, George E, Scambler PJ, Brown DT, et al (2006) Hyperdynamic plasticity of chromatin proteins in pluripotent embryonic stem cells. Dev Cell 10: 105–116. 16399082 10.1016/j.devcel.2005.10.017 PMC1868458

[48] Hager GL, McNally JG, Misteli T (2009) Transcription dynamics. Mol Cell 35: 741–753. 19782025 10.1016/j.molcel.2009.09.005 PMC6326382

[49] Thiriet C, Hayes JJ (2006) Histone dynamics during transcription: exchange of H2A/H2B dimers and H3/H4 tetramers during pol II elongation. Results Probl Cell Differ 41: 77–90. 16909891 10.1007/400_009

[50] Bhattacharya D, Talwar S, Mazumder A, Shivashankar GV (2009) Spatio-temporal plasticity in chromatin organization in mouse cell differentiation and during Drosophila embryogenesis. Biophys J 96: 3832–3839. 19413989 10.1016/j.bpj.2008.11.075 PMC3297759

[51] Kimura H (2005) Histone dynamics in living cells revealed by photobleaching. DNA Repair (Amst) 4: 939–950. 15905138 10.1016/j.dnarep.2005.04.012

[52] Kimura H, Cook PR (2001) Kinetics of core histones in living human cells: little exchange of H3 and H4 and some rapid exchange of H2B. J Cell Biol 153: 1341–1353. 11425866 10.1083/jcb.153.7.1341 PMC2150718

[53] Fan JY, Gordon F, Luger K, Hansen JC, Tremethick DJ (2002) The essential histone variant H2A.Z regulates the equilibrium between different chromatin conformational states. Nat Struct Biol 9: 172–176. 11850638 10.1038/nsb767

[54] Thambirajah AA, Li A, Ishibashi T, Ausio J (2009) New developments in post-translational modifications and functions of histone H2A variants. Biochem Cell Biol 87: 7–17. 19234519 10.1139/O08-103

[55] Fan JY, Rangasamy D, Luger K, Tremethick DJ (2004) H2A.Z alters the nucleosome surface to promote HP1alpha-mediated chromatin fiber folding. Mol Cell 16: 655–661. 15546624 10.1016/j.molcel.2004.10.023

[56] Seefeldt B, Kasper R, Seidel T, Tinnefeld P, Dietz KJ, et al (2008) Fluorescent proteins for single-molecule fluorescence applications. J Biophotonics 1: 74–82. 19343637 10.1002/jbio.200710024

[57] Flynn RA, Almada AE, Zamudio JR, Sharp PA Antisense RNA polymerase II divergent transcripts are P-TEFb dependent and substrates for the RNA exosome. Proc Natl Acad Sci U S A 108: 10460–10465. 21670248 10.1073/pnas.1106630108 PMC3127934

[58] Rahl PB, Lin CY, Seila AC, Flynn RA, McCuine S, et al c-Myc regulates transcriptional pause release. Cell 141: 432–445. 20434984 10.1016/j.cell.2010.03.030 PMC2864022

[59] Sprague BL, Pego RL, Stavreva DA, McNally JG (2004) Analysis of binding reactions by fluorescence recovery after photobleaching. Biophys J 86: 3473–3495. 15189848 10.1529/biophysj.103.026765 PMC1304253

[60] Rangasamy D, Greaves I, Tremethick DJ (2004) RNA interference demonstrates a novel role for H2A.Z in chromosome segregation. Nat Struct Mol Biol 11: 650–655. 15195148 10.1038/nsmb786

[61] Mavrich TN, Jiang C, Ioshikhes IP, Li X, Venters BJ, et al (2008) Nucleosome organization in the Drosophila genome. Nature 453: 358–362. 18408708 10.1038/nature06929 PMC2735122

[62] Teves SS, Henikoff S (2011) Heat shock reduces stalled RNA polymerase II and nucleosome turnover genome-wide. Genes Dev 25: 2387–2397. 22085965 10.1101/gad.177675.111 PMC3222904

[63] Whittle CM, McClinic KN, Ercan S, Zhang X, Green RD, et al (2008) The genomic distribution and function of histone variant HTZ-1 during C. elegans embryogenesis. PLoS Genet 4: e1000187. 18787694 10.1371/journal.pgen.1000187 PMC2522285

[64] Surface LE, Thornton SR, Boyer LA Polycomb group proteins set the stage for early lineage commitment. Cell Stem Cell 7: 288–298. 20804966 10.1016/j.stem.2010.08.004

[65] Hu G, Cui K, Northrup D, Liu C, Wang C, Tang Q, Ge K, Levens D, Crane-Robinson C, Zhao K (2013) H2A.Z Facilitates Access of Active and Repressive Complexes to Chromatin in Embryonic Stem Cell Self-Renewal and Differentiation. Cell Stem Cell 12: 1–13. 23260488 10.1016/j.stem.2012.11.003 PMC3570599

[66] Cuadrado A, Corrado N, Perdiguero E, Lafarga V, Munoz-Canoves P, et al (2010) Essential role of p18Hamlet/SRCAP-mediated histone H2A.Z chromatin incorporation in muscle differentiation. Embo J 29: 2014–2025. 20473270 10.1038/emboj.2010.85 PMC2892367

[67] Fazzio TG, Huff JT, Panning B (2008) An RNAi screen of chromatin proteins identifies Tip60-p400 as a regulator of embryonic stem cell identity. Cell 134: 162–174. 18614019 10.1016/j.cell.2008.05.031 PMC4308735

[68] Papamichos-Chronakis M, Krebs JE, Peterson CL (2006) Interplay between Ino80 and Swr1 chromatin remodeling enzymes regulates cell cycle checkpoint adaptation in response to DNA damage. Genes Dev 20: 2437–2449. 16951256 10.1101/gad.1440206 PMC1560417

[69] Papamichos-Chronakis M, Watanabe S, Rando OJ, Peterson CL (2011) Global regulation of H2A.Z localization by the INO80 chromatin-remodeling enzyme is essential for genome integrity. Cell 144: 200–213. 21241891 10.1016/j.cell.2010.12.021 PMC3035940

[70] Wu WH, Alami S, Luk E, Wu CH, Sen S, et al (2005) Swc2 is a widely conserved H2AZ-binding module essential for ATP-dependent histone exchange. Nat Struct Mol Biol 12: 1064–1071. 16299513 10.1038/nsmb1023

[71] Watanabe S, Radman-Livaja M, Rando OJ, Peterson CL (2013) A histone acetylation switch regulates H2A.Z deposition by the SWR-C remodeling enzyme. Science 340: 195–199. 23580526 10.1126/science.1229758 PMC3727404

[72] Draker R, Ng MK, Sarcinella E, Ignatchenko V, Kislinger T, et al (2012) A combination of H2A.Z and H4 acetylation recruits Brd2 to chromatin during transcriptional activation. PLoS Genet 8: e1003047. 23144632 10.1371/journal.pgen.1003047 PMC3493454

[73] Halley JE, Kaplan T, Wang AY, Kobor MS, Rine J Roles for H2A.Z and its acetylation in GAL1 transcription and gene induction, but not GAL1-transcriptional memory. PLoS Biol 8: e1000401. 20582323 10.1371/journal.pbio.1000401 PMC2889906

[74] Kim HS, Vanoosthuyse V, Fillingham J, Roguev A, Watt S, et al (2009) An acetylated form of histone H2A.Z regulates chromosome architecture in Schizosaccharomyces pombe. Nat Struct Mol Biol 16: 1286–1293. 19915592 10.1038/nsmb.1688 PMC2788674

[75] Krogan NJ, Baetz K, Keogh MC, Datta N, Sawa C, et al (2004) Regulation of chromosome stability by the histone H2A variant Htz1, the Swr1 chromatin remodeling complex, and the histone acetyltransferase NuA4. Proc Natl Acad Sci U S A 101: 13513–13518. 15353583 10.1073/pnas.0405753101 PMC518788

[76] Valdes-Mora F, Song JZ, Statham AL, Strbenac D, Robinson MD, et al Acetylation of H2A.Z is a key epigenetic modification associated with gene deregulation and epigenetic remodeling in cancer. Genome Res 22: 307–321. 21788347 10.1101/gr.118919.110 PMC3266038

[77] Kalocsay M, Hiller NJ, Jentsch S (2009) Chromosome-wide Rad51 spreading and SUMO-H2A.Z-dependent chromosome fixation in response to a persistent DNA double-strand break. Mol Cell 33: 335–343. 19217407 10.1016/j.molcel.2009.01.016

[78] Sarcinella E, Zuzarte PC, Lau PN, Draker R, Cheung P (2007) Monoubiquitylation of H2A.Z distinguishes its association with euchromatin or facultative heterochromatin. Mol Cell Biol 27: 6457–6468. 17636032 10.1128/MCB.00241-07 PMC2099601

[79] Boyer LA, Plath K, Zeitlinger J, Brambrink T, Medeiros LA, et al (2006) Polycomb complexes repress developmental regulators in murine embryonic stem cells. Nature 441: 349–353. 16625203 10.1038/nature04733

[80] Beard C, Hochedlinger K, Plath K, Wutz A, Jaenisch R (2006) Efficient method to generate single-copy transgenic mice by site-specific integration in embryonic stem cells. Genesis 44: 23–28. 16400644 10.1002/gene.20180

[81] Iuchi S, Dabelsteen S, Easley K, Rheinwald JG, Green H (2006) Immortalized keratinocyte lines derived from human embryonic stem cells. Proc Natl Acad Sci U S A 103: 1792–1797. 16446420 10.1073/pnas.0510953103 PMC1413677

[82] Marson A, Levine SS, Cole MF, Frampton GM, Brambrink T, et al (2008) Connecting microRNA genes to the core transcriptional regulatory circuitry of embryonic stem cells. Cell 134: 521–533. 18692474 10.1016/j.cell.2008.07.020 PMC2586071

[83] Bendall SC, Hughes C, Stewart MH, Doble B, Bhatia M, et al (2008) Prevention of amino acid conversion in SILAC experiments with embryonic stem cells. Mol Cell Proteomics 7: 1587–1597. 18487603 10.1074/mcp.M800113-MCP200 PMC2556023

[84] Thomas CE, Kelleher NL, Mizzen CA (2006) Mass spectrometric characterization of human histone H3: a bird's eye view. J Proteome Res 5: 240–247. 16457588 10.1021/pr050266a

[85] Sherman MKaNE (2000) Protein Sequencing and Identification Using Tandem Mass Spectrometry Sons JWa, editor: John Wiley and Sons.

[86] Garcia BA, Mollah S, Ueberheide BM, Busby SA, Muratore TL, et al (2007) Chemical derivatization of histones for facilitated analysis by mass spectrometry. Nat Protoc 2: 933–938. 17446892 10.1038/nprot.2007.106 PMC4627699

[87] Jaffe JD, Keshishian H, Chang B, Addona TA, Gillette MA, et al (2008) Accurate inclusion mass screening: a bridge from unbiased discovery to targeted assay development for biomarker verification. Mol Cell Proteomics 7: 1952–1962. 18534968 10.1074/mcp.M800218-MCP200 PMC2559937

[88] Goshima G, Kiyomitsu T, Yoda K, Yanagida M (2003) Human centromere chromatin protein hMis12, essential for equal segregation, is independent of CENP-A loading pathway. J Cell Biol 160: 25–39. 12515822 10.1083/jcb.200210005 PMC2172742

[89] Toyoda Y, Yanagida M (2006) Coordinated requirements of human topo II and cohesin for metaphase centromere alignment under Mad2-dependent spindle checkpoint surveillance. Mol Biol Cell 17: 2287–2302. 16510521 10.1091/mbc.E05-11-1089 PMC1446084

