# Supplementary figures and images for: H2A.Z Acidic Patch Couples Chromatin Dynamics to Regulation of Gene Expression Programs during ESC Differentiation
Source: PLoS Genet. 2013 Aug 22;9(8):e1003725. doi: 10.1371/journal.pgen.1003725 (PMC3749939; doi:10.1371/journal.pgen.1003725)

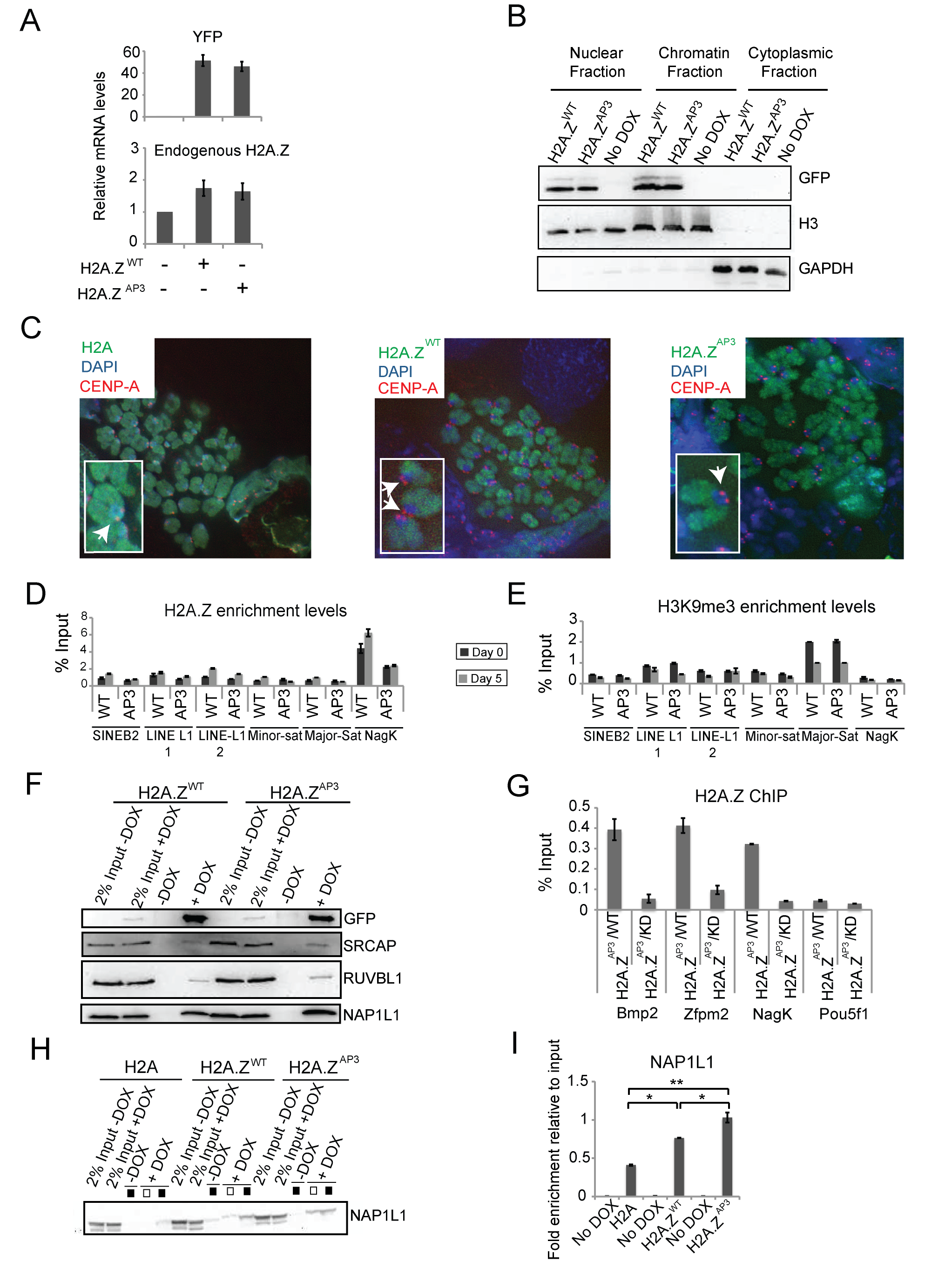

Supplement: Figure S2 — H2A.Z acidic patch mutant is capable of chromatin incorporation. (A) Real-time PCR showing comparable transcript levels of H2A.ZWT and H2A.ZAP3 mutant transgene in the presence of endogenous H2A.Z. (B) Representative western blot showing the enrichment of nuclear, chromatin, and cytoplasmic fractions isolated from H2A.ZWT, H2A.ZAP3, and uninduced (No DOX) ESCs. GFP antibodies were used to probe for the presence of H2A.ZWT and H2A.ZAP3 transgenes in the various cellular fractions. H3 antibodies were used as a marker for nuclear and chromatin fractions and GAPDH was used as a cytoplasmic marker. (C) Chromosome spreads generated from nuclei isolated from H2A.ZWT, H2A.ZAP3 and H2A stained with DAPI (blue) and centromeric H3 variant-CENP-A (red) suggest that H2A.ZAP3 expression shows a similar distribution pattern as H2A.ZWT, indicating the ability of H2A.ZAP3 to effectively incorporate into DNA in chromosomes. The green fluorescence signal is from the YFP-fused transgene expression (as indicated above each image). Quantitative PCR on ChIP DNA generated using GFP (D) and H3K9me3 (E) antibodies in H2A.ZWT (WT) and H2A.ZAP3 (AP3) ESCs and Day 5 RA-differentiated. LINE-L1-1 and 2 refers to primers directed against ORF1 and ORF2 respectively of the LINE-L1 repetitive elements. (F) Co-immunoprecipitation analyses with GFP reveals by western blot that both H2A.ZWT and H2A.ZAP3 interact with H2A.Z-specific incorporation module- SRCAP, ATP-dependent DNA helicase RUVBL1 (a subunit of chromatin remodeling complexes INO80, Tip60-p400 and SRCAP) and histone chaperone Nap1, suggesting that the reduced enrichment of H2A.ZAP3 genome-wide is not due to its inability to associate with its incorporation complex. (G) ChIP qPCR on DNA immuno-precipitated with H2A.Z antibodies (Abcam, ab4174) in cells over expressing H2A.ZAP3 (for more than four passages), in the presence of endogenous H2A.Z (H2A.ZAP3/WT) show normal H2A.Z localization compared to reduced enrichment of H2A.ZAP3 expressing ES [file pgen.1003725.s002.tif]
